# Drop Friction and Failure on Superhydrophobic and Slippery Surfaces

**DOI:** 10.1021/acsnano.5c01142

**Published:** 2025-05-14

**Authors:** Abhinav Naga, Liam R. J. Scarratt, Chiara Neto, Periklis Papadopoulos, Doris Vollmer

**Affiliations:** † Institute for Multiscale Thermofluids, School of Engineering, 3124 The University of Edinburgh, Edinburgh EH9 3FD, United Kingdom; ‡ University of New South Wales College, Sydney 2052, Australia; § School of Chemistry, 4334 The University of Sydney, Sydney 2006, Australia; ∥ University of Sydney Nano Institute, The University of Sydney, Sydney 2006, Australia; ⊥ Department of Physics, 37796 University of Ioannina, Ioannina GR-45110, Greece; # University Research Center of Ioannina, Institute of Materials Science and Computing, Ioannina GR-45110, Greece; ¶ Physics at Interfaces, 28308 Max Planck Institute for Polymer Research, 55128 Mainz, Germany

**Keywords:** drops, wetting, friction, adhesion, capillarity, surface cleaning, interfacial phenomena, lubrication, roughness

## Abstract

The mobility of drops on a surface influences how much water and energy is required to clean the surface. By controlling drop mobility, it is possible to promote or reduce fogging, icing, and fouling. Superhydrophobic and slippery liquid-infused surfaces both display high drop mobility despite being ‘lubricated’ by fluids having very different viscosities. Superhydrophobic surfaces rely on micro- and/or nanoscale textures to trap air pockets beneath drops, minimizing solid–liquid contact. In contrast, on liquid-infused surfaces, these solid textures are filled with an immiscible liquid lubricant. Over the past few years, innovations in experimental and computational methods have provided detailed new insights into the static and dynamic wetting properties of drops on these surfaces. In this review, we describe the criteria needed to obtain stable wetting states with low drop friction and high mobility on both surfaces, and discuss the mechanisms that have been proposed to explain the origins of friction on each surface. Drops can collapse from the low-friction Cassie state to the high-friction Wenzel state on both surfaces, but the transition follows different pathways: on liquid-infused surfaces, the wetting ridge near the drop edge plays a central role in triggering collapse, a phenomenon not observed on superhydrophobic surfaces. This review emphasizes that a liquid-infused surface cannot be simply viewed as a superhydrophobic surface with the air pockets replaced by lubricant. The wetting ridge surrounding drops on liquid-infused surfaces significantly alters most of the drop’s properties, including macroscopic shape, friction mechanisms, and the mechanism of collapse to a Wenzel state.

## Graphical abstract

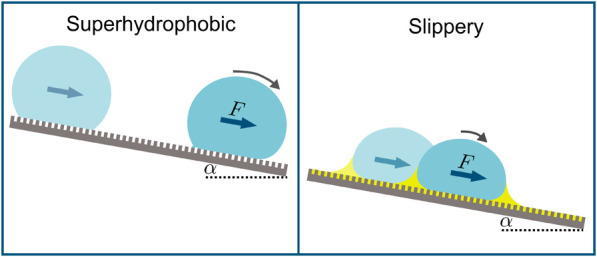

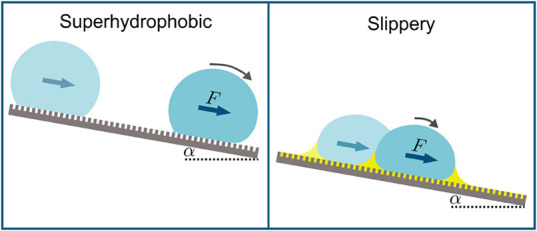

## 1 Introduction

The field of wetting has attracted the interest of scientists and engineers for decades due to its combination of scientific curiosity and practical importance.^1–3^ In the natural world, wetting of surfaces by liquids can be a matter of survival for several plants, birds, and insects.^4,5^ For instance, water repellency is essential for birds and insects to maintain air cushions in feathers or wings and thus maintain vital thermal insulation and the ability to fly. Plants have also evolved strategies to flourish in a variety of conditions. For example, the lotus leaf, which has inspired the field of superhydrophobicity, is self-cleaning: water drops (*e.g*., from rain) collect dirt particles and pathogens from the leaf as they roll off, allowing the plant to remain clean and disease-free.^6^ Over the past two decades, researchers have designed, fabricated and modeled a variety of surfaces with antifouling, anti-icing,^7–16^ and easy-to-clean properties,^17–20^ several of which were inspired by natural surfaces (see Figure 1 for a timeline and refs. 21–24 for reviews on the various types of bioinspired surfaces). In particular, superhydrophobic surfaces and liquid-infused surfaces have attracted great interest due to their remarkably low friction against liquid drops,^25^ with over 10,000 papers published on these surfaces over the past 5 years, i.e. 2020–2024.^168^

**Figure 1:**
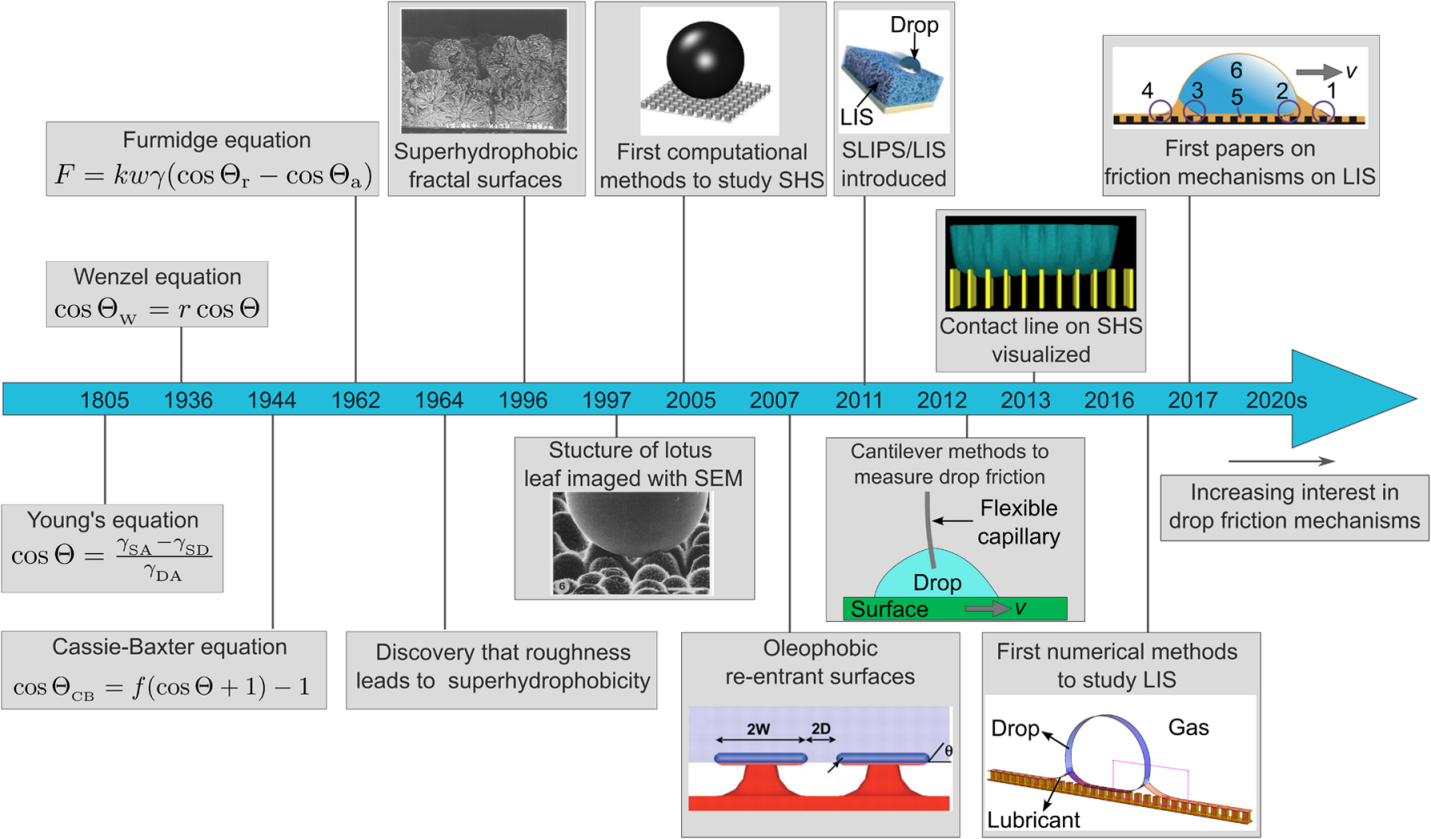
Timeline showing some key discoveries and selected methods to study the wetting of SHS and LIS. Although Young’s equation is named after Thomas Young and attributed to his 1805 paper,^34^ the equation was first written by Dupré in 1869.^35,36^ The equations used to describe the wetting of rough surfaces were pioneered by Wenzel^37^ as well as Cassie and Baxter.^38^ Dettre and Johnson observed that a hydrophobic surface can become superhydrophobic by increasing its roughness.^39^ In the 1990s, the first fractal-like superhydrophobic surfaces were fabricated in a lab (1996 inset. Reproduced from ref 40. Copyright 1996 American Chemical Society) and scanning electron microscopy was adopted to image the structure of natural superhydrophobic surfaces such as the lotus leaf (1997 inset. Reproduced with permission from ref 6. Copyright 1997 Springer Nature).^6,40^ Computational methods were developed to explicitly simulate drop interaction with superhydrophobic surfaces in the early 2000s (2005 inset. Reproduced with permission from ref 41. Copyright 2005 American Chemical Society.).^41^ Re-entrant structures were designed in 2007 (2007 inset. From ref 42, Tuteja et al. 2007. Reprinted with permission from AAAS) to repel liquids having interfacial tension below 0.03 N/m, such as octane.^42^ The concept of LIS was first mentioned (as hemisolids) in a 2008 review paper by Quéré.^26^ LIS became popular in 2011 (2011 inset. Reproduced with permission from ref 30. Copyright 2011 Springer Nature).^27,30^ Cantilever methods were introduced to measure drop friction in 2012 (2012 inset. Schematic inspired by ref 43).^43^ Several variations of these methods have been introduced since.^44–46^ The contact line of drops collapsing to the Wenzel state on SHS was visualized with interference microscopy in 2007 and with confocal microscopy in 2013 (2013 inset. Reproduced from ref 47. Copyright 2013 National Academy of Sciences).^47,48^ The first numerical methods to study drop dynamics on LIS were developed in 2016 (2016 inset. Reproduced from ref 49. Copyright 2018 American Chemical Society),^49,50^ and the first papers discussing friction on LIS were published in 2017 (2017 inset. Reprinted figure with permission from ref 56. Copyright 2020 by the American Physical Society).^44,51^

The aim of this review is to discuss the latest progress in the fundamental understanding of the wetting of dry and lubricated nano/microstructured surfaces. We focus on two of the most widely studied super liquid-repellent surfaces, namely superhydrophobic surfaces (SHS) and liquid-infused surfaces (LIS). In the literature, liquid-infused surfaces are also referred to as hemisolids,^26,27^ lubricant-infused surfaces,^28^ lubricant-impregnated surfaces,^29^ and slippery liquid-infused porous surfaces (SLIPS).^30^ Other types of superliquid repellent surfaces have also been fabricated, for example nanometer thin liquid-like or slippery omniphobic covalently attached liquid (SOCAL) surfaces.^31,32^ A detailed discussion of SOCAL surfaces is beyond the scope of this review (see ref^33^ for comparison between SOCAL and LIS and ref^25^ for a comparison between SOCAL, SHS, and LIS).

In this review, we describe the wetting states that drops adopt on SHS and LIS, discuss the mechanisms that give rise to friction when drops move on these surfaces, and compare the pathways via which drops may collapse from a low-friction Cassie state to a Wenzel state with significantly higher friction. Since water is the most common target liquid used in fundamental studies, we use the term ‘superhydrophobic’ (super water-repellent) and consider water as the main liquid to be repelled in this review. However, most of the discussions also extend to nonaqueous drops on superoleophobic (super oil-repellent) and superamphiphobic (super repellent to both aqueous and oily liquids) surfaces.

A superhydrophobic surface is a rough hydrophobic surface with micro and/or nanotextures. Drops rest on top of the textures, making only partial contact with the solid.^26,52–55^ Liquid-infused surfaces are similar to superhydrophobic surfaces in terms of solid structure, but the porous or micro/nano structured surface is imbibed with a liquid lubricant.^26–30,44,51,56,57^ The geometry of the solid texture plays a key role for both SHS and LIS, determining the ability to keep air pockets or lubricant trapped in the structure. On SHS, the geometry and surface energy of the solid structure determine whether the surface can repel only water or whether lower surface tension liquids (*e.g*., organic solvents) can also be repelled.^42,58,59^ On LIS, the lubricant must be chemically compatible with the surface, spontaneously imbibe the solid, and be immiscible with the target liquid being repelled.^44,60–62^ For fundamental studies, typical choices of lubricant include silicone oils and mineral oils because they are available in a wide range of viscosities for almost the same surface tension (for a review on types of lubricants used, see refs. 44,62 ).

LIS have fundamentally different static and dynamic wetting properties compared to SHS because of the formation of a lubricant wetting ridge around the drop.^28–30,49,56,57,63^ Due to the wetting ridge, a LIS cannot be simply considered as a SHS with the air pockets replaced by lubricant. Replacing the air by lubricant would correspond to having a drop on a surface that is fully submerged in lubricant. Only 2 fluids would be involved (drop and lubricant). In contrast, on LIS, 3 fluids are involved (drop, lubricant and air). The presence of 3 fluids is significantly more complex to image experimentally and model computationally. In section 2 on “Wetting states with low friction and variety of solid structures”, we describe the criteria required to obtain a wetting state with low friction on SHS and LIS and highlight a selection of surface geometries that can be used to fabricate SHS and LIS. A vast variety of surface geometries are available in the literature. Here, we focus on a few representative examples to highlight the benefits of different categories of structures.

Over the last years, our understanding of friction between drops and SHS/LIS has been transformed by the widespread adoption of new methods to measure forces^43–45,64–67^ and to image the interaction between drops and surfaces.^28,44,46,55,57^ With the development of cantilever-based techniques to move drops, it has become possible to probe the steady-state friction force of drops moving at very low speeds on SHS,^25,68–71^ and image the reorganization of lubricant when drops move on LIS.^44,57,72^ These techniques have led to significant progress in our understanding of drop dynamics, leading to new physical mechanisms being discovered over a decade after the discovery of SHS and LIS. In section 3 on ‘Friction mechanisms’, we discuss the latest progress on the friction and energy dissipation mechanisms when drops move on SHS and LIS, with the aim of highlighting the fundamental differences between the two surfaces.

Drops only experience low friction when the contact between the drop and solid surface is minimized. However, the drop can invade the surface texture when the pressure in the liquid exceeds the critical pressure the structure can withstand.^47,73–76^ An increase in the drop pressure can occur due to mechanical compression, drop impact, or due to the increase in curvature (Laplace pressure) as drops evaporate. When a drop collapses in the gaps between the solid textures, it typically sticks strongly to the surface. The surface fails and can no longer repel the drop easily. In section 4 on ‘Collapse of the low friction states’, we discuss and compare the pathways via which drops collapse from a Cassie state with low friction to a Wenzel state with significantly higher friction.

## 2 Wetting States with Low Friction and Variety of Solid Structures

### 2.1 Wetting of Flat Surfaces

The wettability of an ideal flat surface with respect to a liquid drop is generally characterized by the equilibrium contact angle, Θ_e_, between the liquid and the surface according to Young’s law,^34^

1 $$\cos\Theta_{e}=\frac{\gamma_{SA}-\gamma_{SD}}{\gamma_{DA}}$$

Here, *γ*_*SA*_, *γ*_*SD*_, and *γ*_*DA*_ are the solid–air, solid–liquid, and liquid–air interfacial tensions, respectively. For a rigid solid, the solid–air interfacial tension *γ*_*SA*_ is equivalent to the surface energy of the solid (with respect to air). The term surface tension is often used instead of interfacial tension if one of the phases is air. Equation 1 is valid for ideal surfaces that are assumed to be atomically flat, homogeneous, nondeformable, nonreactive, nonadaptive, and highly conductive. Chemical or topographical inhomogeneities cause pinning of the three-phase contact line, where the contact line is the locus of points where all three phases (drop, air, solid) meet. Due to contact line pinning, the measured contact angle always differs from its equilibrium value. On nonconducting surfaces, contact line pinning also arises because moving liquid–air interfaces deposit electrostatic charges on the surface.^77^ Furthermore, van der Waals or electric double layer forces may deform the free liquid surface in the very close vicinity of the three-phase contact line (below around 100 nm). These deformations also affect the contact angle measured by traditional contact angle goniometers, which we term the ‘material contact angle’ in the following.^78,79^ Due to these complexities, the ideal surfaces that are required for Young’s law to be strictly valid have not been achieved yet. Consequently, in practice, a sessile drop can take every angle between the so-called advancing and receding contact angles, Θ_*a*_ and Θ_*r*_, respectively.

The advancing contact angle is defined as the maximum contact angle reached just before the three-phase contact line starts advancing when the drop is expanded by pumping more liquid into it. Analogously, the receding contact angle is the minimum value reached when liquid is pumped out of the drop and the three-phase contact line starts receding. The difference between the advancing and receding contact angle is termed contact angle hysteresis, ΔΘ = Θ_*a*_ – Θ_*r*_.^78,80–83^ It should be noted that in general both the advancing and receding angles depend on the velocity of the respective contact line. The difference between the angle at the front and rear of a drop on a tilted surface just before the onset of motion is sometimes also used to calculate contact angle hysteresis. Contact angle hysteresis is important for many applications because it is related to the friction force between the drop and the surface, as will be explained in detail in Section 3. Local microscopic variations in the wettability of the surface can be characterized by mapping variations in the friction force or normal adhesion when a small droplet is scanned across the surface.^84,85^

According to eq 1, the contact angle depends on the interfacial tensions, *γ*_*SA*_, *γ*_*SD*_, and *γ*_*DA*_. These interfacial tensions can become functions of time, *γ*_*SA*_(*t*), *γ*_*SD*_(*t*), and *γ*_*DA*_(*t*), if the drop reacts with the surface or if the surface changes its composition or properties due to the presence of a drop.^86^ The most common method to tune the wettability of a surface is by changing *γ*_*SA*_ by chemically modifying the surface. Since a higher contact angle leads to a smaller solid/liquid contact area, it is often desirable to reduce the surface energy, *γ*_*SA*_, of the solid (since a more negative numerator in eq 1 leads to a higher contact angle). Yet, even with the lowest surface energy coating available (fluorinated hydrocarbons), the maximum water contact angle that can be obtained on a flat surface does not exceed around 120^◦^.^87,88^

### 2.2 Wetting of Rough Surfaces and Superhydrophobicity

To achieve contact angles larger than 120^◦^ on the macroscopic scale, the surface must be rough. As early as 1944, Cassie and Baxter recognized that when a drop is deposited on a surface with nano- and micrometre roughness, a stable layer of air may become trapped below the drop, leading to contact angle values higher than predicted by the Young’s equation and increasing the ease with which the drop rolls off the surface.^38^ In 1964, Dettre and Johnson observed that the advancing and receding contact angles on a hydrophobic substrate suddenly increased when the roughness exceeded a critical value, as shown in Figure 2a.^39^ Below the critical value, the drop makes full contact with the solid. Above the critical value, air pockets remain trapped between the rough structures. These air pockets prevent the drop from making full contact with the solid, leading to a so-called Cassie–Baxter state, also known as a Cassie state. In the 1990s, Nienhuis and Barthlott used scanning electron microscopy to image a variety of biological specimens and concluded that the self-cleaning properties of the naturally superhydrophobic Lotus leaf result from drops resting partially on trapped air pockets.^5,6,89^ This discovery inspired numerous researchers to mimic the natural world when designing functional surfaces with anti-icing, anti­(bio)­fouling, or easy-to-clean properties.

**Figure 2:**
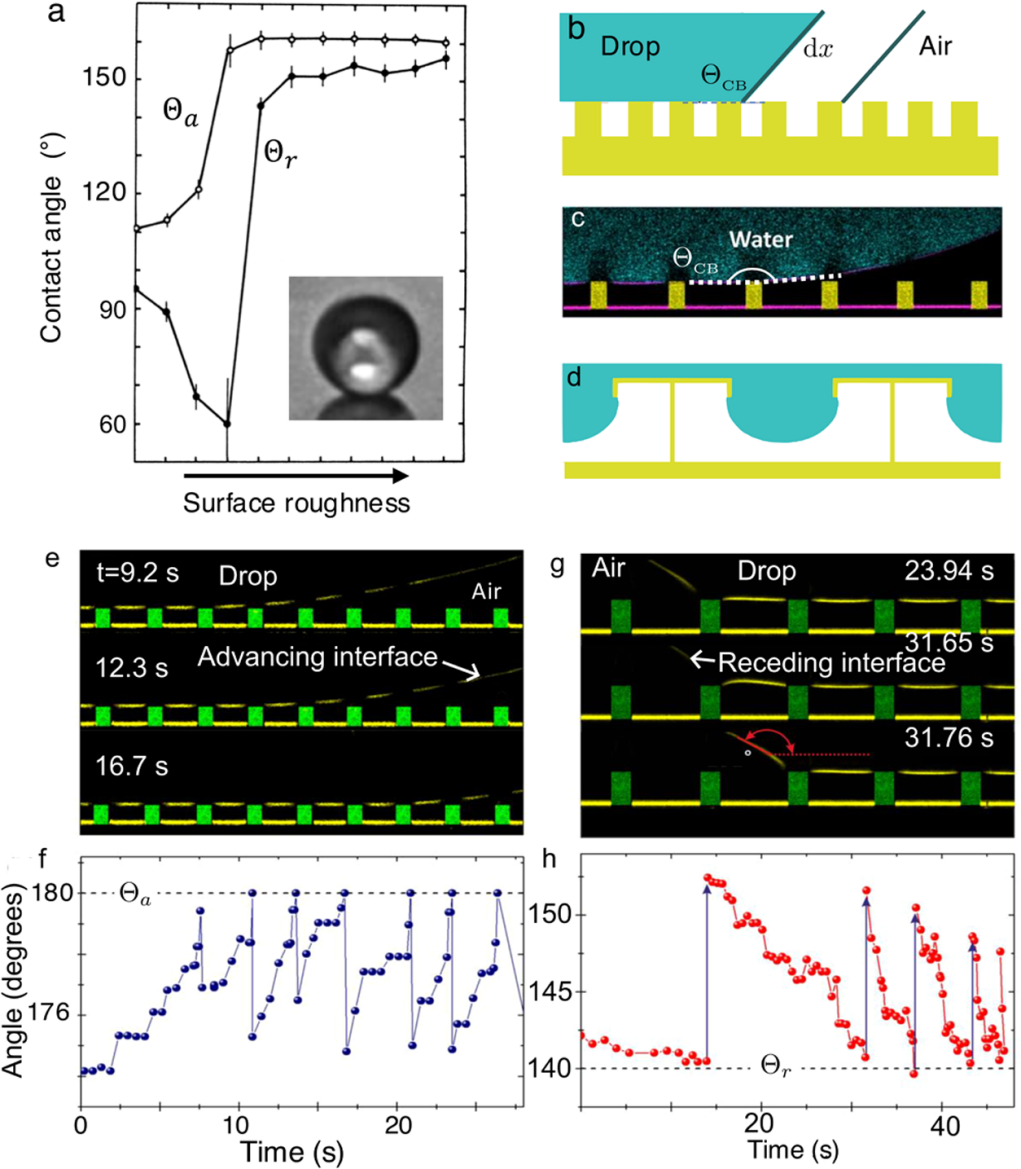
Advancing and receding contact angles on rough surfaces. (a) The apparent advancing and receding contact angles increase beyond a certain roughness, indicating a transition to the Cassie state. Adapted from ref 39. Copyright 1964 American Chemical Society. Note that the apparent receding contact angle initially decreases with roughness, as predicted by the Wenzel equation (eq 15) when the contact angle is less than 90°, suggesting that the drop is initially in a Wenzel state until a critical roughness is reached. (b) Sketch of how a drop (greenish blue) advances on a micropillar array (yellow). Here it is assumed that the drop-air interfaces are flat. (c) Confocal microscopy image of a water drop on a micropillar array. Fluorescently labeled water is shown in cyan, pillars are drawn in yellow, and reflection at the drop-air interface is shown in magenta. Pillar center-to-center (pitch) distance is equal to *p* = 40 μm. (d) Re-entrant structures such as the T-shape structures shown here can be used to repel liquids with surface tension below 0.03 N/m. Adapted with permission under a Creative Commons CC BY 4.0 License from ref 90. Copyright 2019 The American Association for the Advancement of Science. (e) Advancing side of a water drop on micropillars imaged by confocal microscopy. Reflection at the air/water interface is shown in yellow and pillars are drawn in green. There is negligible sagging of the air–water interface between the pillars because the drop radius is much larger than the spacing of the pillars. The sagging depth scales as δ ∼ *p* ^2^/*R*, where *p* is the pitch distance of the pillars and *R* is the drop radius. Here, the pitch distance is 20 μm and the drop radius is around 1 mm, which gives a sagging depth of δ ∼ 0.4 μm. Such a small sagging depth cannot be observed with the resolution of the images. (f) The apparent advancing contact angle extracted from the profiles in (e) is close to 180°. Abrupt changes correspond to the advancement of the drop to the next row of pillars. (g) Receding side of a water drop. Pillar center-to-center distance is equal to 40 μm. Apparent receding contact angles defined at the height shown in red are plotted in (h). Panels (c, e–h) adapted with permission ref 55. Copyright 2016 by the American Physical Society.

For many years, the modeling of static contact angles on superhydrophobic surfaces focused on an equilibrium thermodynamic description. For an ideal rough surface, a thermodynamic energy balance can be used to determine whether the drop partially rests on an air cushion or fully wets the protrusions.^38,53,54^ Here, the total energy is dominated by the interfacial energies. As sketched in Figure 2b, the displacement of the contact line by a distance d*x* is accompanied by the formation of fresh liquid/solid and liquid/vapor surface area. For a drop deposited on a homogeneous micropillar array, the displacement of the contact line is associated with a change in the interfacial energy per unit length given by^26,54^

2 $$dE_{\mathrm{CB}}=f\left(\gamma_{\mathrm{SD}}-\gamma_{\mathrm{SA}}\right)\;dx+\left(1-f\right)\gamma_{\mathrm{DA}}\;dx+\gamma_{\mathrm{DA}}\cos\Theta_{\mathrm{CB}}\;dx$$

Here, *f* is the solid fraction, defined as the ratio of the solid area exposed to the drop to the projected area and Θ_CB_ is the Cassie–Baxter contact angle between the drop on the micropillar array, as defined in Figure 2b,c.

Analogously, the interfacial energy per unit length for a fully wetted (Wenzel state) micropillar array is given by,

3 $$dE_{W}=\left(\gamma_{\mathrm{SD}}-\gamma_{\mathrm{SA}}\right)r\;dx+\gamma_{\mathrm{DA}}\cos\Theta_{W}\;dx$$

where *r* is the roughness factor, defined as the ratio between the total surface area of the solid and projected contact area.

By comparing the energy associated with having air pockets under the drop (first two terms on the right of eq 2) with the energy associated with the drop fully wetting the gaps between the micropillars (first term on the right of eq 3) we can deduce that air pockets are favored when the roughness factor of the surface exceeds,^26^

4 $$r>\left(1-f\right)\frac{\gamma_{\mathrm{DA}}}{\gamma_{\mathrm{SD}}-\gamma_{\mathrm{SA}}}+f$$

The inequality in eq 4 provides a quantitative explanation to the critical roughness at which Dettre and Johnson observed a sudden increase in contact angle (Figure 2a).

By setting d*E*_CB_ = 0 in eq 2 (i.e., thermodynamic equilibrium), we obtain an expression for the apparent contact angle when the drop rests on top of the pillars,

5 $${\cos\;\Theta }_{\mathrm{CB}}=f\left(\cos\Theta_{e}+1\right)-1$$

where Θ_e_ is the Young’s contact angle defined on a flat surface made of the same material as the rough surface. Equation 5 is called the Cassie–Baxter equation. Since the Cassie–Baxter equation was derived based on thermodynamic arguments it gives the equilibrium apparent contact angle on SHS and does not include contact angle hysteresis. In the following we use the term ‘apparent contact angle’ when referring to the contact angle on a rough surface to distinguish it from the material contact angle on a flat surface.

The derivation of the Cassie–Baxter equation assumes a surface with homogeneous roughness, such that the solid fraction can be defined independently of where precisely the drop is positioned on the surface. However, not all parts under the drop influence the apparent contact angle equally. Gao and McCarthy showed that only the region in the close vicinity of the three-phase contact line determines the apparent contact angle.^91^ Thus, for surfaces with nonuniform roughness, the Cassie–Baxter equation can still be used, provided that the solid fraction is defined as the local value appropriate to the drop perimeter.^92^ However, on rough surfaces pinning of the three-phase contact line cannot be ignored, even in an ideal case, because roughness inherently introduces topographical heterogeneities. In addition, contact line pinning can still arise from the usual mechanisms that are also relevant on a real flat surface: chemical heterogeneities, adaptation of the surface to the presence of the liquid, or charging of the surface by impacting or sliding drops. Consequently, on rough surfaces, the true energy landscape can be much more complex than the one used to derive the Cassie–Baxter equation.

The Cassie–Baxter equation cannot explain the fact that even the best superhydrophobic surfaces have to be tilted by at least a few degrees before drops begin to roll off. A roll-off angle larger than zero implies that the angle at the front (advancing side) of the drop is greater than that at the rear (receding side) instead of being given by the single value provided by the Cassie–Baxter equation. A suggested guideline to determine whether a drop is in a superhydrophobic state (i.e., a Cassie state with low drop friction), is to check that the apparent receding contact angle exceeds 140° and that small (10 μL) drop rolls off the surface at tilt angle of less than 10°.

Most liquids have a surface tension lower than that of water. Lower surface tension tends to translate to lower local equilibrium contact angles. This makes the superhydrophobic state less stable or even unstable. However, with appropriate surface geometries, drops can still rest on an air cushion even when the material’s contact angle is less than 90°. For example, geometries with overhangs can enhance pinning of the contact line (Figure 2d).

On SHS, it is difficult to accurately measure the apparent contact angle using traditional contact angle goniometers due to difficulties in determining the position of the three-phase contact line with sufficient accuracy.^67,93^ To gain insight into the movement of the three-phase contact line on superhydrophobic surfaces, more sophisticated techniques are required. Using laser scanning confocal microscopy, Schellenberger et al. showed that when a drop moves on a superhydrophobic surface, the three-phase contact line at the front of the drop remains pinned onto the edge of a protrusion until the drop touches the next protrusion (Figure 2e). The three-phase contact line does not advance downward along the pillars side walls, because the material’s contact angle remains below the advancing contact angle and sagging of the interface is negligible. As soon as the drop touches the next protrusion, the leading contact line jumps forward and the process repeats. This implies that locally, the apparent advancing contact angle is typically close to 180° (Figure 2f).

At the rear of the drop, the contact line remains pinned, causing the capillary bridge between the drop and the protrusion to stretch until the apparent contact angle decreases to the apparent receding contact angle (Figure 2g,h). Then, the contact line jumps forward, accompanied by a relaxation of the capillary bridge, before the process repeats. This discontinuous motion is characteristic of superhydrophobic surfaces. Superhydrophobic surfaces can show an apparent contact angle hysteresis of more than 40° because of the large apparent advancing contact angle.

### 2.3 Liquid-Infused Surfaces

In this review, we focus on the original version of liquid-infused surfaces as introduced by Wong et al. (2011) and Lafuma and Quéré (2011).^27,30^ A LIS is a micro/nano structured solid surface that is imbibed with lubricant. The solid and lubricant must be chemically compatible. Chemical compatibility means that the material contact angle the lubricant forms with the solid surface should be lower than the critical contact angle for spontaneous wicking.^94^ The lubricant may locally dewet the substrate when a drop is placed on the surface. Other definitions of LIS are still under debate. For example, it was proposed that the lubricant under investigation should form a thermodynamically stable film even in the presence of a drop,^44^ which requires a positive spreading parameter *S* between the lubricant and the substrate in the presence of both in air and under the drop.^29^ The spreading parameter *S* is related to the interfacial tensions according to *S* = *γ*_SD_ – *γ*_SL_ – *γ*_DL_. In our opinion this definition of LIS is impractical because with this definition, the stability of the lubricant sensitively depends on the choice of the liquid drop, which would mean that whether a surface classifies as a LIS or not would depend on the choice of the liquid drop. Other varieties of liquid-infused surfaces exist, including those without a rough or porous substrate, such as lubricated polymer brushes or swollen silicone surfaces. The static and dynamic properties of drops on these various types of liquid-infused surfaces can be fundamentally different and are beyond the scope of this review (see the review by Hauer et al. (2024)^33^ for a comparison of different lubricated surfaces).

Wetting of LIS follows the same concepts as wetting of SHS, but with a few important differences. The apparent contact angle and the drop-lubricant interface can only be observed using techniques that allow us to distinguish between the drop and lubricant, such as fluorescence microscopy or confocal microscopy. Apparent contact angle hysteresis is typically much smaller on LIS, and a low roll-off angle can still be obtained even though the macroscopic contact angle measured using traditional contact angle goniometers is typically much smaller (∼100°–110°). In the following we use the term macroscopic contact angle for drops on LIS if the angle was measured using traditional contact angle goniometers to distinguish it from the apparent contact angle of the drop-lubricant interface, as seen within the lubricant meniscus. Furthermore, the consequences of chemical heterogeneities of the solid surface on the wetting state are less serious as the lubricant rearranges and spreads to cover defects.

Although the thermodynamic modeling of LIS follows similar concepts as the modeling of SHS, theoretical models need to be extended to consider the lubricant. This makes the behavior much richer as pointed out by Smith et al.^29^ The lubricant can either wet or dewet the drop-solid interface and can also spread over the drop (Figures 3a,b).^29^ Another complication is that the volume of lubricant can be hard to control in practice in order to achieve a perfect filling of the rough or porous solid. Surfaces can thus be underfilled, perfectly filled, or overfilled. Underfilling, perfect filling, and overfilling means that the lubricant level is below, at, and above the height of the solid structures, respectively. To increase the stability of the lubricant, hierarchical structures have been designed (Figure 3c).

**Figure 3:**
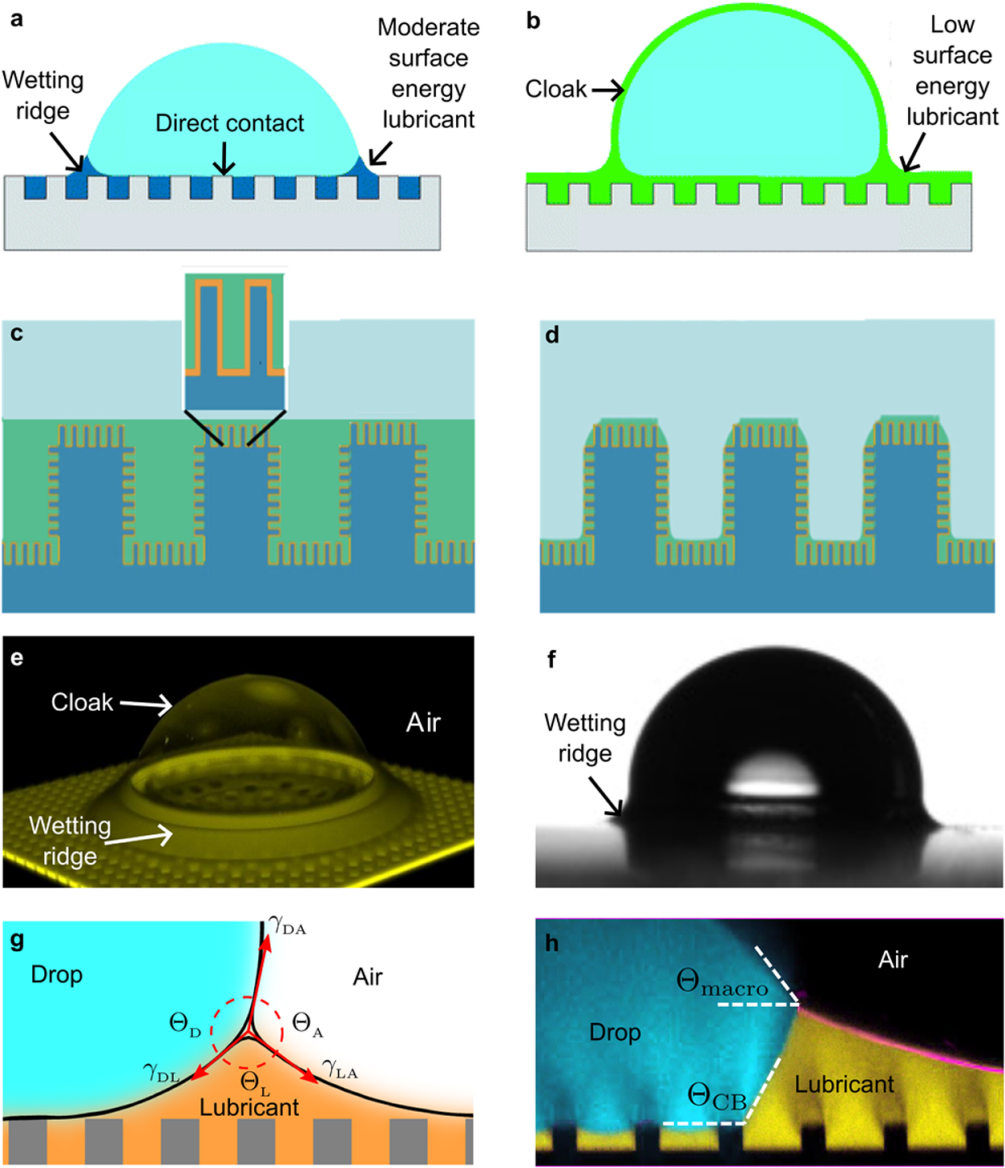
Drops on LIS. (a) Drop deposited on liquid-infused micropillar array. The lubricant fills the gaps but dewets from the pillars top face and from the drop. (b) The lubricant wets the pillars and cloaks the drop. Panels (a) and (b) reproduced with permission from ref 29. Copyright 2013 by the Royal Society of Chemistry. (c) Sketch of a LIS with a hierarchical structure submerged in water, for example under a drop (light blue). The silicone micropillars (dark blue) are covered with nanostructures. The nanostructure is chemically modified (orange) to ensure good chemical compatibility with the lubricant (green). The inset shows an enlarged view of the infiltrated nanostructure. (d) An example of a micro-Wenzel but nano-Cassie state. The thickness of the lubricant layer separating the drop from the substrate is determined by the height of the infused structure. Panels (c) and (d) adapted from ref 95. Copyright 2015 American Chemical Society. (e) Confocal image of a drop on a micropillar array. Only the lubricant is fluorescently labeled. The drop is surrounded by a wetting ridge and covered by a lubricant cloak. Adapted with permission under a Creative Commons CC BY 4.0 License from the Supporting Information of ref 57. Copyright 2024, The Authors. (f) Backlight microscopy (goniometer) image of a drop deposited on a liquid-infused micropillar array. (g) The Neuman triangle and the contact angles at the air-drop-lubricant contact line. Adapted from ref 49. Copyright 2018 American Chemical Society. (h) Confocal image of the wetting ridge surrounding the drop. Fluorescently labeled water in cyan, lubricant in yellow, reflection at the lubricant/air interface in magenta. The macroscopic contact angle Θ_macro_ is smaller than the apparent contact angle Θ_CB_ at the drop-lubricant-solid contact line. Panels (f) and (h) adapted with permission under a Creative Commons CC BY 3.0 License from ref 28. Copyright 2015 by the Royal Society of Chemistry.

Even if the drop penetrates the microstructure (micro-Wenzel state), the high capillary pressure in the nanostructures can keep the lubricant in place, ensuring that the water remains in a nano-Cassie state (Figure 3d). The drop can still have high mobility as long as the liquid-infused nano-Cassie state remains (Figure 3d).

A key feature of drops on lubricated surfaces is the wetting ridge. When a drop is deposited on the surface, excess lubricant underneath the drop is pushed outward. The vertical component of the interfacial tension of the drop pulls the lubricant up in the vicinity of the three-phase contact line.^96,97^ A wetting ridge forms (Figure 3e,f). The wetting ridge is made up of lubricant from underneath the drop and the surrounding region (away from the drop). The shape and height of the wetting ridge depend on the interfacial tensions, the amount of lubricant available, and the capillary force retaining the lubricant in the solid textures. It is not possible to fully resolve the shape of the wetting ridge using a traditional camera with backlighting because of the lack of contrast between the drop and the lubricant, as shown in Figure 3f. Numerical simulations (Figure 3g) or fluorescence microscopy (Figure 3e,h) are required to resolve the interface between the drop and the wetting ridge.

The lubricant can also spread over (‘cloak’) the drop to lower the drop-air interfacial tension, *γ*_*DA*_ (Figure 3e). Whether the cloak is thermodynamically stable depends on the interplay of the interfacial tensions and the disjoining pressure between the drop/lubricant and lubricant/air interfaces. A cloak forms when the drop-lubricant-air interfacial tension *γ*_*DA*_ > *γ*_*DL*_ + *γ*_*LA*_, where *γ*_*DA*_ is the interfacial tension of the drop/air interface (without cloak), *γ*_*DL*_ is the interfacial tension drop-lubricant interface and *γ*_*LA*_ is the interfacial tension of the lubricant/air interface. Typically, the thickness of the cloak is in the order of 20 nm.^28^ However, when the drop is in motion, more lubricant may be dragged over the drop, causing the cloak to thicken.^72^

When the lubricant does not cloak the drop, the Neumann angles (shown in Figure 3g) at the tip of the wetting ridge are determined by considering the balance of interfacial tensions at the drop/lubricant/air three-phase contact line. The Neumann angles are related to the interfacial tensions according to the relation,^98^

6 $$\frac{\gamma_{\mathrm{DA}}}{\sin\Theta_{L}}=\frac{\gamma_{\mathrm{DL}}}{\sin\Theta_{A}}=\frac{\gamma_{\mathrm{LA}}}{\sin\Theta_{D}}$$

where Θ_*L*_, Θ_*A*_, and Θ_*D*_ are the angles measured in the lubricant, air, and drop phase, respectively. When the drop is cloaked, the interfacial tension of the pure drop/air interface *γ*_*DA*_ must be replaced by the interfacial tension of the cloaked interface, which is given by γ_LA_ + γ_DL_ to a first order approximation. When the drop evaporates or moves, the Neuman triangle can rotate but the angles remain unaltered to the point where the drop becomes so small that van der Waals interactions become important.^99^

The Cassie–Baxter equation (eq 5) is still used to estimate the apparent contact angle between the solid and the drop/lubricant interface on LIS. As in the case of superhydrophobic surfaces, the Cassie–Baxter equation cannot describe the pinning of the contact lines. Furthermore, the apparent contact angle is hidden by the wetting ridge and cannot be measured using traditional contact angle goniometers. Due to these difficulties, a macroscopic contact angle is usually defined at the tip of the wetting ridge or by fitting a circular arc to the drop and extrapolating the angle where the arc meets the substrate (Figure 3f).^98,100^ This macroscopic angle measured in this way is typically much smaller than the equilibrium apparent contact angle Θ_CB_ predicted by the classical Cassie–Baxter equation (Figure 3h). The latter is shown in detail in Figure 4. On LIS, the apparent advancing angle is close to 180° (Figure 4a,b), as is the case for SHS (Figure 2). The apparent receding contact angle (Figure 4c,d) depends on whether the top of the pillars remains coated with lubricant or whether the drop makes contact with the substrate, Figure 4d. For both the advancing and receding cases, the evolution of the contact angles closely resembles those on SHS, Figure 2f,h.

**Figure 4:**
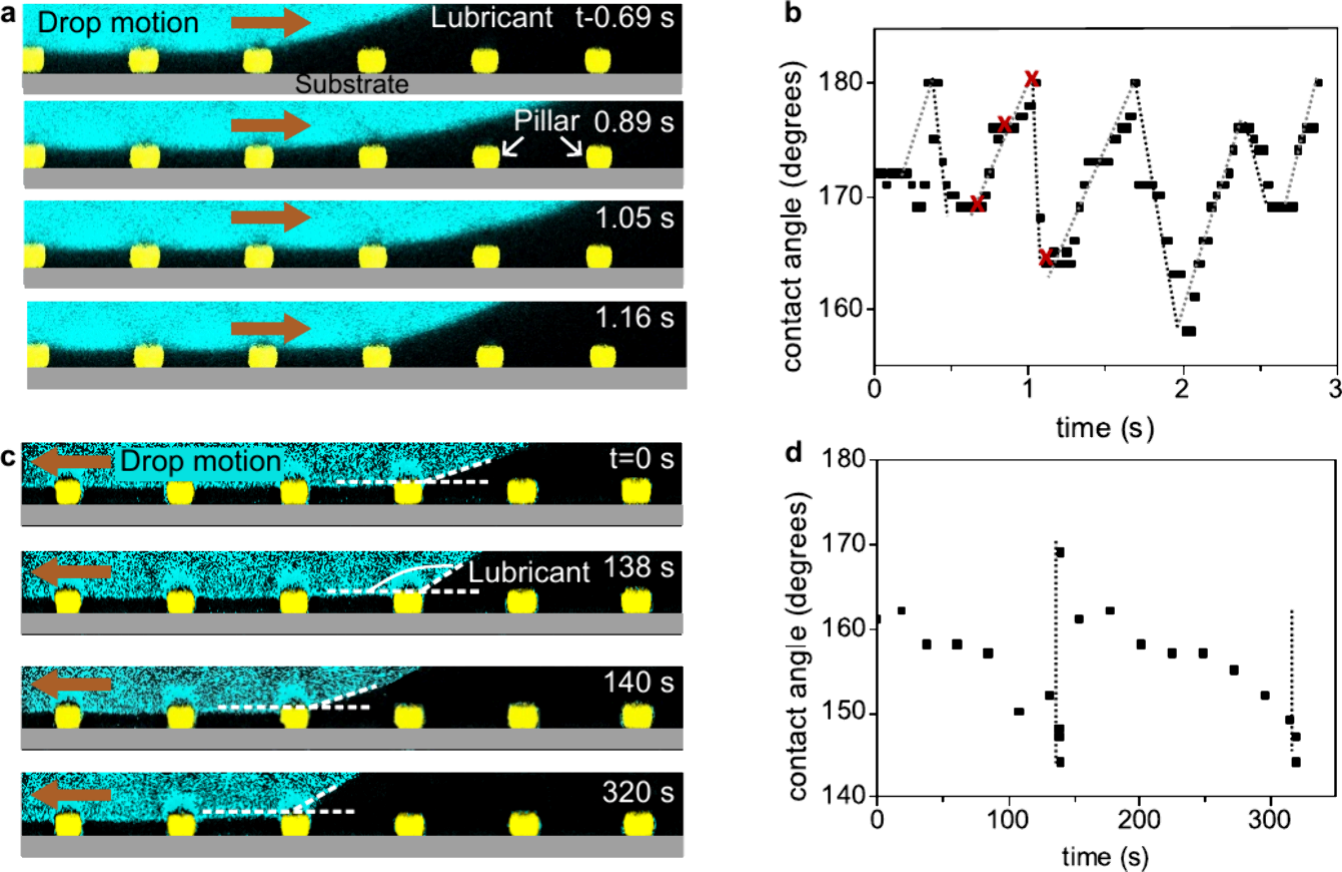
Apparent advancing and receding contact angles at the solid-drop-lubricant contact line on LIS. (a) Confocal microscopy images monitoring the advancement of a drop (cyan) on a LIS (yellow pillars). The drop makes contact with the pillars. The lubricant (FC70) was not dyed and appears black. The drop advanced by applying air flow. Pillars are drawn by hand, because they appear shorter than their real height in the confocal microscope, due to refractive index mismatch between the pillars and lubricant. The height of the pillars was determined using scanning electron microscopy. (b) The apparent advancing contact angle continuously increased until it reached 180° and subsequently decreased to a lower value abruptly. The contact angles were extracted from the movie from which the snapshots in (a) were taken. The red crosses correspond to the snapshots in (a). (c) Confocal microscopy images of the receding side of a drop on LIS. The drop receded due to evaporation. (d) The apparent contact angle at the receding side continuously decreased until it approached the apparent receding contact angle. All panels adapted with permission under a Creative Commons CC BY 3.0 License from ref 28. Copyright 2015 by the Royal Society of Chemistry.

### 2.4 Solid Geometries

The chemistry and geometry of the solid structure play a key role in controlling the static and dynamic properties of drops on both SHS and LIS. The solid structure of SHS and LIS can span several orders of magnitude in length scale, from several nanometers to hundreds of micrometres. Scanning electron microscopy is the most commonly used technique to image the smallest features (nanometres to micrometres). However, due to its poor vertical resolution it is not possible to accurately determine a 3D-height profile of rough surfaces. Atomic force microscopy can also be used to obtain the topography of the surface with a lateral spatial resolution of the order of a few nanometres. However, the finite size of the tip prevents mapping deep and narrow pores. For larger structures (few micrometres and above), optical microscopy or cameras with high-resolution macro lenses can be sufficient. So far, no technique exists that can fully resolve nanoporous or fractal like-surfaces, but an effective surface area can still be estimated if the pores are open.

A stable SHS requires a stable air cushion under a deposited drop. Therefore, the surface energy of the solid substrate should be as low as possible. This can be achieved by using hydrophobic substrates, such as polyethylene, polysiloxane, or Teflon. However, most untreated substrates are naturally hydrophilic. In this case, the surface energy can be lowered by chemical modification of the surface by silanization with siloxanes or fluorinated molecules. The surface energy can be as low as 0.004 J/m^2^ for perfluorinated compounds.^101^

The stability of the Cassie state can also be tuned by tuning the surface geometry. Almost all hydrophobic surfaces can be made superhydrophobic by increasing the roughness, *e.g*. a smooth Teflon coating can be applied on a shrinkable substrate to achieve hierarchical micro- and nanostructured wrinkles.^102^ Some ways to further increase the roughness include covering the surface with particle aggregates (Figure 5a), coating the surface with fibers (Figure 5b), or by chemically etching the substrate. Particle aggregates have the advantage that they can be applied quickly and easily. However, they are brittle because the necks connecting the network of particles are thin and fragile. Fibers or filaments benefit from their flexibility, reducing the likelihood of breakage of the connecting necks. Etching can result in coatings with particularly low solid fractions (Figure 5c). Particle aggregates, filaments and chemically etched surfaces all have some degree of randomness. For fundamental studies, model surfaces with regular patterns are often preferred (Figure 5d–i). Model surfaces are typically fabricated using lithography, which allows fine-tuning of the shape, height, spacing, and solid fraction of micron-sized structures.

**Figure 5:**
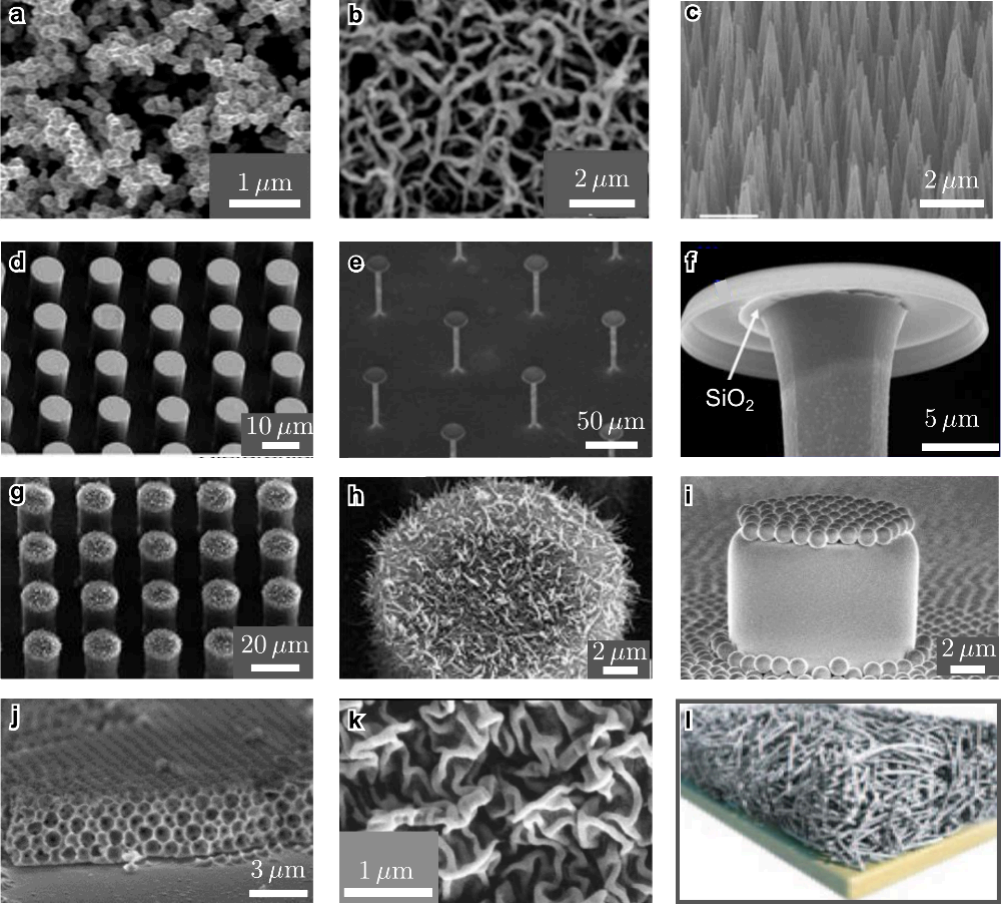
Solid geometries used for SHS and LIS. All panels correspond to images taken using scanning electron microscopy, except for panel (l), which is a schematic. (a) Candle soot nanoparticles covered by silica. To lower the surface energy, the hydrophilic silica shells were coated with a semifluorinated silane. Adapted with permission from ref 105. Copyright 2012, American Association for the Advancement of Science. (b) Silicone nanofilaments. Adapted from ref 106 (modified the scale bar). Copyright 2016 WILEY-VCH Verlag GmbH & Co. KGaA, Weinheim. (c) Black silica with micrometer-sized spikes. Adapted with permission under a Creative Commons CC BY 4.0 License from ref 107. Copyright 2023 Springer Nature. (d) Silica microposts with wax layer. (e–f) Silica microposts with doubly re-entrant nano-overhangs. (g–h) Hierarchical structure of silicone micropillars with Lotus wax tubules. Panels (d), (g), (h) adapted with permission from ref 108. Copyright 2009 by the Royal Society of Chemistry. Panels (e) and (f) adapted with permission from ref 58. Copyright 2014, American Association for the Advancement of Science. (i) Janus micropillars, covered with a layer of colloidal particles. Adapted with permission under a Creative Commons CC BY 3.0 License from ref 109. Copyright 2014 by the Royal Society of Chemistry. (j) Inverse opal. Adapted with permission under a Creative Commons License CC BY 3.0 from ref 28. Copyright 2015 by the Royal Society of Chemistry (k) Teflon wrinkles, reprinted with permission under a Creative Commons License CC BY 4.0 from ref 102. Copyright 2022 Springer Nature. (l) Schematic of a functionalized porous solid for LIS. Reprinted with permission from ref 30. Copyright 2011, Springer Nature Limited.

Transparent samples are required when imaging the drop from below to obtain detailed insights on the shape and reorganization of the three-phase contact line. Transparent micropillar arrays can be fabricated using photolithography. Advanced lithography also allows the fabrication of micropillars with overhangs or micropillars with so-called doubly re-entrant overhangs, which are crucial to achieve oil-repellency (Figure 5e,f). To reduce the solid fraction of model structures, the top face of protrusions can also be roughened (Figure 5g), resulting in hierarchical roughness or two-tier structures. For example, micropillars can be covered with fiber-like structures (Figure 5h) or with particles (Figure 5i). Various other strategies have been proposed to tune and optimize the geometry of coatings for different potential applications or purposes.^103^ Recent developments in 3D printing technology, such as two-photon polymerization, have also made it possible to print 3D structures even down to 100 nm resolution.^104^ However, for 3D printed surfaces to be superhydrophobic without needing additional treatment or coating, the printing material must be inherently hydrophobic, which is often not the case.

For several applications, in addition to having an air cushion under the drop, it is also important that the superhydrophobic coating resists damage due to mechanical shear. While it is currently not possible to produce structures that fully resist mechanical damage, there are ways to minimize the consequences of mechanical damage on drop friction. For example, the low-friction property of the coatings can be prolonged by using self-similar multilayered coatings such that a superhydrophobic layer remains even when the topmost layer is abraded. Microstructures have been combined with nanostructures in arrangements that increase mechanical durability while maintaining high water repellency, such as the so-called armored arrangement proposed by Wang et al.^110^ Polyester textiles coated with silicone nanofilaments (Figure 5b) keep their superhydrophobicity until the hydrophobic fabric is almost fully destroyed by scratching with sandpaper or a metal sponge.^10^ Even when silicone nanofilaments on the fibers’ tops face are eroded by scratching, the fibers’ sides can remain intact. This prevents the drop from wetting the fabric.

On LIS, mechanical damage to the nano/microstructure is less critical because the lubricant can reorganize to cover defects. However, LIS suffer from lubricant depletion, which can lead to significant degradation of their slippery property. The range of solid geometries that can be used to make LIS is even broader than that to prepare SHS. Geometries that can be used for LIS include all the SHS geometries as well as additional types of geometries, such as honeycomb structures (Figure 5j), Teflon wrinkles (Figure 5k), porous structures (Figure 5l), and structures with holes. In practice, it can be harder to make SHS with low static friction than LIS with low static friction because more care is required to ensure there are no imperfections that can act as pinning sites for the drop on SHS. On LIS, the lubricant spreads on the solid to cover up imperfections. Therefore, all geometries that work for SHS can in principle also work for LIS, but not all geometries that work for LIS will make a good SHS. For example, structures with holes (Figure 5j) or dense porous structures can have a very high solid fraction, which would lead to strong pinning if they are used as superhydrophobic surfaces. But they can work well for LIS because the high solid fraction can enhance the solid’s ability to retain lubricant, thus reducing reduc depletion. Oil repellency can be achieved on LIS without overhangs. To repel oil, the oil should not dewet the lubricant from the solid. Ideally the spreading parameter of the lubricant on the solid should be positive both under air and under the oil. When choosing a solid geometry for LIS, we need to consider the ease of filling the structure with lubricant to ensure surfaces can be imbibed easily as well as the ability of the structure to retain the lubricant to slow down the rate of depletion of lubricant.

Typical choices of lubricant include silicone oils, mineral oils, and fluorinated oils such as Krytox.^62^

Practically, the main challenge of LIS is that over time, lubricant gets depleted from the surface. There are several mechanisms by which depletion of lubricant can occur, including gravitational drainage, passage through an air/water interface and removal by drops falling off the surface.^111^ Even in the absence of external factors, lubricant depletion can still occur as lubricants typically have low surface tensions and thus spread on the side/bottom walls of substrates or the platform on which the substrate is positioned during experiments. Viscous lubricants deplete at a slower rate but they also lead to a lower drop speed. To reduce depletion of lubricant, the pore size of the coating should be as small as possible. The capillary force holding the lubricant in place scales inversely with the gap size, so nanostructured surfaces lead to better lubricant retention.

## 3 Friction Mechanisms

The homogeneity of a surface, the distribution of pinning sites on a surface, and the ease with which drops roll off a surface can be characterized by measuring the friction force between the drop and the surface. Sometimes, it is also advantageous to measure the normal adhesion force between a droplet and the surface. This can be done using tensiometers^112^ or more accurate techniques such as scanning droplet adhesion microscopy.^85,113^ Scanning droplet adhesion microscopy can provide a spatial resolution down to 10 μm and has been applied to study droplet adhesion on superhydrophobic surfaces. Drops have significantly higher adhesion on LIS due to the much larger contact area than on SHS.^114^ In the following, we will focus on drop friction, on which progress has been remarkable.

### 3.1 Techniques To Measure Drop Friction

Drop friction can be divided into static friction and kinetic friction. Static friction is the force required to initiate the motion of the drop.^65^ Static friction is sometimes also called retention force or lateral adhesion, but recent publications tend to favor the term static friction. Static friction can be caused by pinning/depinning by inhomogeneities on the surface,^115^ contact-line friction due to thermal activation of liquid molecules near the contact line,^116^ electrostatic retardation induced by slide electrification,^77^ and surface adaptation.^86^ Kinetic friction is the force opposing motion once the drop has started moving. It can be due to all the effects that cause static friction, but also due hydrodynamic dissipation,^117^ and aerodynamic resistance.^118^ Typically, the static friction is higher than the kinetic friction, but this is not always the case.^65,119^ In particular, it was shown that the static friction becomes almost equal to the kinetic friction when the initial static drop shape is set to the shape that the drop adopts during motion.^120^

Historically, drop friction has been quantified based on measurements of contact angle hysteresis. Popular methods include measuring the advancing and receding contact angles as liquid is pumped or extracted from a drop (a low droplet inflation/deflation rate of around 0.05 μL per second is typically used) or measuring the angle at the front and rear sides of a drop that is either about to move or already moving in steady-state on a tilted substrate. An alternative is to measure the contact angle when a plate is plunged and extracted from a liquid bath (Wilhelmy plate tensiometry). In the early 2010s, new techniques were developed to measure the friction and adhesion forces between millimeter-sized drops and surfaces directly (see review by ref 67 for details of the various methods). At present, the two common methods that are used to probe the friction between a drop and a SHS or LIS are the tilted plate method and the cantilever method.

In the tilted plate method, a drop with a defined volume, Ω is placed on a surface that is gradually and slowly tilted until the drop begins moving. The tilt angle, α, corresponding to the onset of motion is then recorded. From this tilt angle, the static friction can then be obtained since at the onset of motion, the static friction is equal to the component of the gravitational force acting parallel to the surface, *F*_*g*_ = ρ_drop_ *g*Ω sinα, where ρ_drop_ is the density of the liquid, *g* is the acceleration due to gravity, and Ω is the drop volume. It is important to report the drop volume because it affects the tilt angle corresponding to the onset of motion. The tilted plate method is popular due to its simplicity and ease of implementation, requiring only a high-resolution camera and an adjustable incline. However, the main limitation of the tilted plate method is that it is difficult to obtain the kinetic friction that drops experience while moving on superhydrophopbic surfaces because drops do not move in steady state after overcoming the static friction. Instead, they accelerate rapidly due to the kinetic friction being significantly smaller than the static friction. In contrast, on lubricated surfaces, drops quickly reach steady state and the tilted-plate method is sufficient to characterize the kinetic friction force.

With the cantilever method,^43,65,67^ the drop is placed on a surface and kept in place by an elastic cantilever with a known spring constant. Then, the surface is moved at constant velocity. As the surface moves, the cantilever keeps the drop in position by exerting an equal and opposite force to the friction force between the drop and the surface. During this process, the cantilever deflects by an amount proportional to the force that it exerts on the drop. The friction force can be obtained by measuring the deflection of the cantilever and using Hooke’s law, *F* = *kx*, where *k* is the spring constant of the cantilever and *x* is the deflection.^43,44,65^ The spring constant of the cantilever is typically obtained either by applying a known force to the cantilever and measuring the corresponding deflection or by measuring the natural frequency of oscillations of the cantilever. The cantilever method enables us to measure both the static friction and the kinetic friction force at a wide range of constant velocities on both SHS and LIS. Care must be taken to ensure that the cantilever does not deform the drop significantly and does not reduce the contact area of the drop by lifting it upward, especially on SHS where there is very little normal adhesion between the drop and the surface. Some solutions to tackle these issues include functionalizing the cantilever with a hydrophobic coating to minimize spreading of the drop on the cantilever or pinning the drop to the bottom edge of the cantilever by introducing a sharp gradient in the wettability. Although the cantilever may deform the drop, results obtained with the cantilever method are consistent with those obtained with the tilted plate method when the force is normalized by the width of the contact perimeter between the drop and the substrate.

While experimental techniques allow us to directly measure the friction force, it is often challenging to obtain the velocity profile inside the drop experimentally. These can be obtained with computational methods. Velocity profiles are important to understand which regions within the drop or lubricant layer (on LIS) dissipate energy. For example, recent simulations using the lattice Boltzmann method have enabled us to visualize the velocity profiles in the drop and lubricant and obtain so termed “heatmaps” for the distribution of viscous dissipation (i.e. heat) on LIS.^57^ With computational methods, it is typically also easier to study the influence of different parameters in isolation to gain a more detailed physical understanding of the different mechanisms leading to friction. In the following discussions, we consider data obtained using both experimental techniques and computational methods.

### 3.2 Drop Motion and Friction on Superhydrophobic Surfaces

Contact angle hysteresis between a drop and a surface controls the onset of motion of the drop. Static friction can be related to contact angle hysteresis by integrating the component of the surface tension in the plane of the solid surface along the three-phase contact line. When this line integral is evaluated at the onset of motion, we obtain

7 $$F_{s}=k\gamma_{\mathrm{DA}}w\left(\cos\Theta_{r}-\cos\Theta_{a}\right)$$

Here, *w* is the width of the base of the drop, and Θ_*r*_ and Θ_*a*_ are the apparent receding and advancing contact angles, respectively. The numerical prefactor *k* encompasses details regarding the shape of the three-phase contact line and how the contact angle varies around the contact line. Typically, *k* ≈ 1. Equation 7 is usually called Furmidge’s equation. It highlights that two ingredients are required to obtain a low static friction: a small drop width and a low contact angle hysteresis. However, it is still possible to achieve a low friction even if one of these quantities is not particularly small as long as the other is very small. For example, although the typical width of drops on LIS is comparable to their corresponding width on most flat hydrophobic surfaces, the friction on LIS is still much lower due to their exceptionally low contact angle hysteresis. In contrast, on SHS the apparent contact angle hysteresis can be as high as 40° due to the large apparent advancing contact angle of 180°, but since the drop width is smaller than on flat hydrophobic surfaces (for the same volume), the static friction is still remarkably low on SHS. Friction on SHS is often of the order of 100 nN (*F*/(γ_DA_ *w*) ∼ 1 or less), which is over 100 times smaller than the gravitational force for a drop radius of 1 mm.

A low static friction is a necessary, but not a sufficient criterion to achieve a high drop mobility. The mobility is related to how fast the drop moves. A high mobility not only requires a small static friction but also a small kinetic friction after the onset of motion. The kinetic friction is the resistive force experienced during motion. Typically, the static friction is smaller on LIS, but the kinetic friction is smaller on SHS. As we will discuss below, different mechanisms contribute to the kinetic friction on SHS depending on the viscosity of the drop, the geometry of the solid, and the velocity of the drop (Figure 6a). These mechanisms include viscous dissipation due to velocity gradients in the drop (significant when it is viscous, *e.g*., for glycerol drops) (Figure 6b), dissipation in the air wedge in front and behind the drop (Figure 6c), depinning of capillary bridges (Figure 6d), dissipation in the plastron layer and air film between the drop and the surface (Figure 6e), and even aerodynamic resistance at high speeds (Figure 6f).

**Figure 6:**
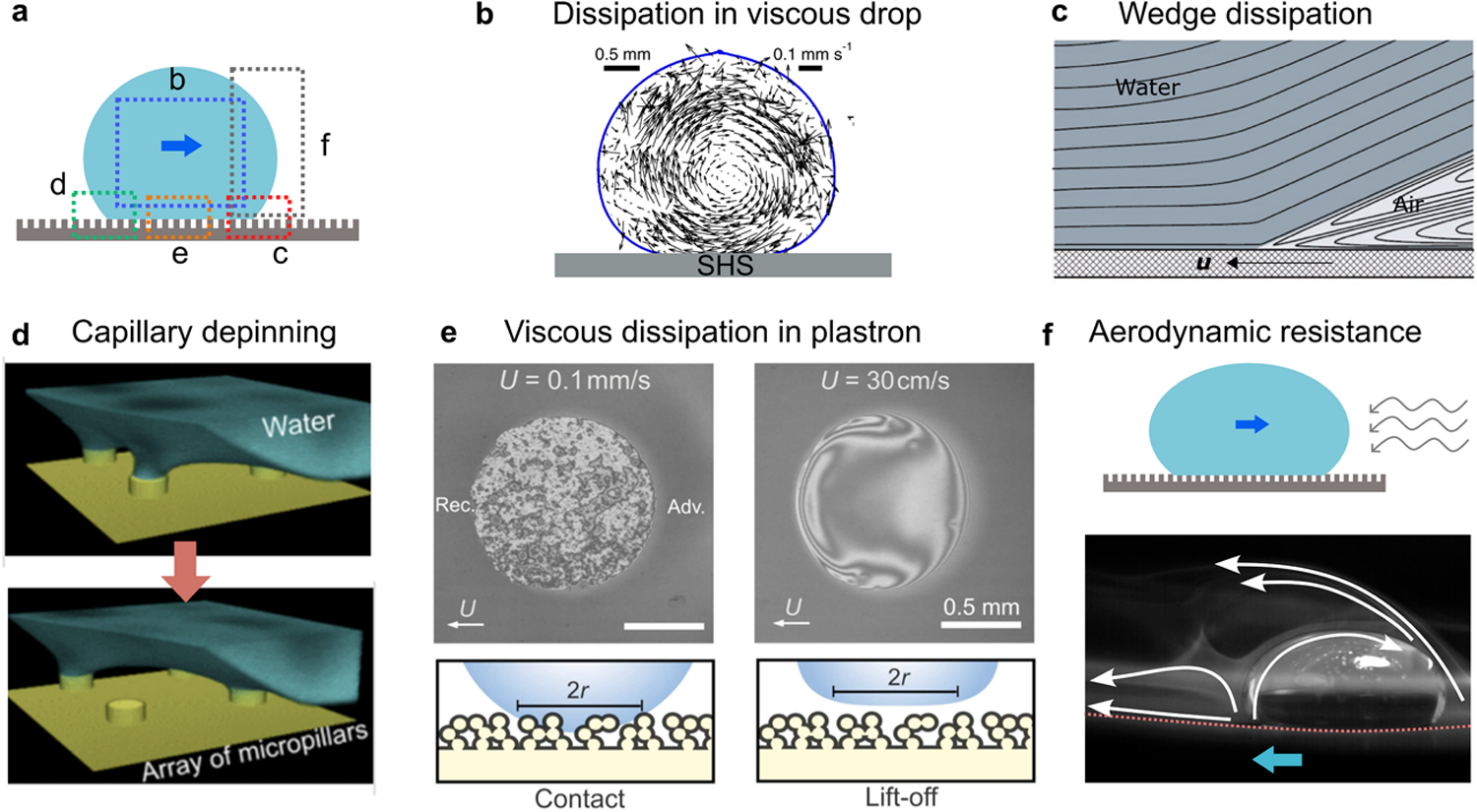
Mechanisms giving rise to friction when drops move on superhydrophobic surfaces. (a) Schematic of a drop moving on a SHS. The different regions where dissipation arises are highlighted by the dotted rectangles. (b) Velocity profile inside a drop showing that flow inside the drop follows a rolling trajectory. The velocity profiles were obtained by imaging the trajectory of tracer particles experimentally. The figure is adapted with permission under a Creative Commons License CC BY 4.0 from ref 68. Copyright 2020 The Authors. The flow inside the drop can lead to significant friction due to viscous shear in the drop when the liquid is viscous. (c) Schematic of velocity streamlines at the advancing wedge close to the drop/air interface. At the wedge, the dissipation in the air phase can exceed that in the drop phase since the flow is strongly confined in the air phase. The panel is adapted with permission from ref 121. Copyright 2013 AIP Publishing. (d) Depinning of a capillary bridge as a drop recedes on an array of micropillars. The image was taken using confocal microscopy. Adapted from ref 122. Copyright 2017 American Chemical Society. (e) Drop base visualized using reflection interference contrast microscopy. At low speeds (0.1 mm/s), the drop is in contact with the surface, but at high speeds (30 cm/s), the drop lifts off and an air film (which gives rise to dissipation) forms between the drop and the solid texture, as shown in the schematics below the interferograms. In these measurements, the drop was held in position by a cantilever while the substrate moved at constant velocity. The direction of the arrows labeled U shows the direction of motion of the substrate. The advancing and receding sides of the drop are labeled as Adv. and Rec., respectively. Reprinted with permission under a Creative Commons License CC BY 3.0 from ref 71. Copyright 2024 by the Royal Society of Chemistry. (f) Water drop rolling inside a superhydrophobic cylinder spinning clockwise at 26 cm/s. To illustrate the flow of air around the drop, we have added the white arrows on top of the original image. The white arrows are deduced by observing the flow of air in the movie. Adapted from the supplementary movie in ref 118 under a standard PNAS License.

Drops moving on tilted superhydrophobic surfaces typically accelerate under the action of gravity until they reach a steady state when the friction force balances the gravitational force. For water drops in the superhydrophobic state, the steady state is only reached when the speed reaches several centimeters per second.^118^ This implies that in order to measure friction in steady state at lower speeds, the cantilever method must be used. The dominant mechanisms that lead to friction depends on drop speed. At low speeds (<1 mm/s), friction is dominated by depinning of capillary bridges at the rear side of the drop. Energy must be supplied to extend and rupture these capillary bridges as the drop moves.^122^ Figure 6d shows an example of a capillary bridge depinning at the rear side of a drop. Here, only one capillary bridge is shown but in practice several capillary bridges may depin within a given time interval. Figure 7 shows the normalized friction force as a function of the drop speed. The region at low speeds, where the data follows a horizontal trend, corresponds to the regime where depinning is dominant.

**Figure 7:**
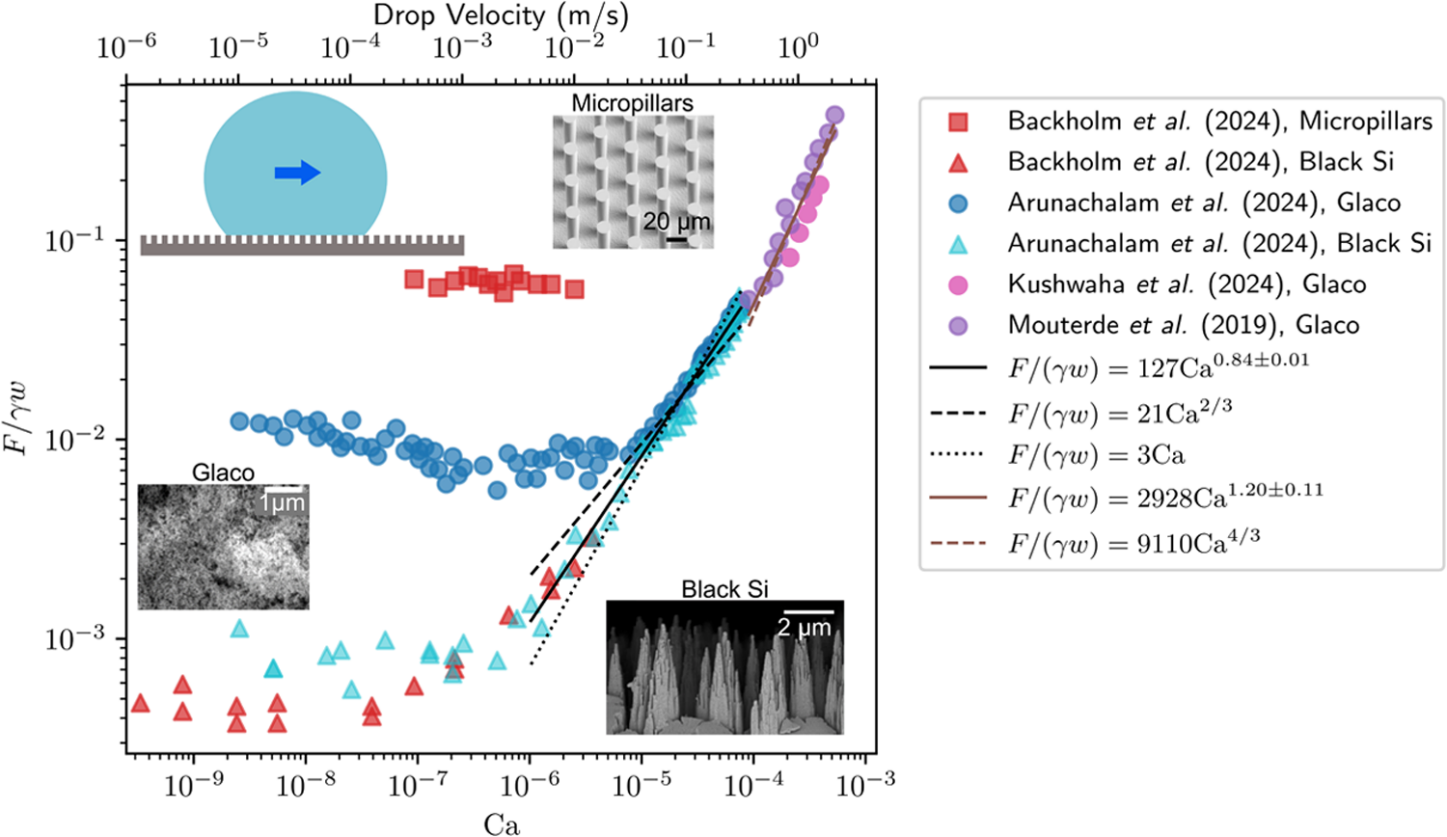
Relationship between friction force, drop velocity, and air capillary number on superhydrophobic surfaces. On the *y*-axis, the friction force is normalized by the product of drop base width and surface tension of the drop. The data is extracted from refs 70, 71, 118, 125, and 126 . The non-normalized friction force spans from tens of nN to several μN. On the *x*-axis, the capillary number is defined in terms of the drop velocity and the air viscosity for all data sets. Throughout this figure, γ denotes the drop-air surface tension. The top *x*-axis shows the drop velocity. In the plot, circle symbols correspond to Glaco-coated substrates, triangles correspond to black silicon, and squares correspond to micropillars. SEM images of some of the surface geometries are included as insets. At low Ca, there is initially a plateau, where the friction force is independent of velocity. In this regime, the friction force is dominated by capillary depinning. The height of this plateau depends on the solid fraction of the surface geometry. Above a critical Ca, the friction becomes velocity-dependent and scales as *F*/(*γw*) ∼ Ca^0.84^ (line of best fit is the solid black line; dashed black line corresponds to a scaling law exponent of 2/3, and dotted black line corresponds to an exponent of 1). In this regime, friction originates predominantly from viscous dissipation in the air layer under the drop. At the highest speeds (Ca > 10^–4^), aerodynamic resistance becomes important and $\frac{F}{\gamma w}∼{\mathrm{Ca}}^{4/3}$ (dashed brown line; solid brown line shows best fit). The micropillar and black silicon inset are reprinted with permission under a Creative Commons License CC BY 4.0 from ref 69. Copyright 2024 Wiley. The Glaco inset is reprinted with permission under a Creative Commons License CC BY 3.0 from ref 71. Copyright 2024 by the Royal Society of Chemistry.

Depinning leads to friction because the surface energy released when capillary bridges depin from a stretched state is quickly lost as heat due to viscous dissipation in the liquid. Had the energy not been dissipated, the elastic energy stored in the capillary bridge would have been converted into kinetic energy to propel the drop forward. Since all the energy is dissipated, the friction force can be obtained from the work required to stretch capillary bridges to their breaking point.^122^ The friction associated with depinning is proportional to the number of capillary bridges and thus depends on the solid fraction, explaining why the different data sets that have a horizontal regime in Figure 7 lie at different positions along the vertical axis.

Several models have been proposed to relate contact angle hysteresis and friction to the solid fraction.^25,35,53,115,123,124^ Reyssat and Quéré modeled the drop/air interface of the depinning capillary bridge as a catenoid and consider how the capillary bridge stretches as it depins to calculate how much energy is stored during this process.^123^ Their model relates contact angle hysteresis to the solid fraction. As shown by Lepikko et al., Reyssat and Quéré’s model can be used in conjunction with the Furmidge equation (eq 7 with *k* = 24/π^3^) to obtain an expression relating the friction force to the solid fraction,^69^

8 $$\frac{F}{\gamma_{DA}w}=\frac{6}{\pi^{3}}\xi \phi \;\ln\frac{\pi }{\phi }$$

where ξ is a parameter related to how strongly the liquid adheres to the solid (e.g., due to surface chemistry) and ϕ is the solid fraction defined as the ratio of the solid area that is in contact with the drop to the projected solid area.

An alternative expression is available from Daniel et al., who propose that $F/\left(\gamma_{DA}w\right)∼\sqrt{\phi }$ .^25^ The relations used by Reyssat and Quéré as well as Daniel et al. to relate contact angle hysteresis to the solid fraction provide excellent fits to experimental data up to ϕ ≈ 0.4,^25^ which is a range that covers most commonly fabricated superhydrophobic surfaces with low friction. The friction associated with depinning has no velocity dependence since the work required to stretch capillary bridges is only a function of the shape of the capillary bridge, with is itself a function of the extension of the capillary bridge but not of the velocity.

Recent experiments show that above a critical speed, the friction force becomes dependent on velocity, as seen in Figure 7. Backhlom et al. proposed that the velocity dependence arises due to viscous dissipation in the air pockets trapped between the solid roughness. They predict that the critical speed above which the friction becomes velocity-dependent is given by $V_{c}∼\frac{4F_{s}h_{p}}{\eta_{\mathrm{air}}\pi w^{2}}$ where *F*_*S*_ is the static friction force associated with the depinning of capillary bridges, η_air_ is the dynamic viscosity of air, and *h*_*p*_ is the height of the solid textures/pillars. The critical speed is higher for taller pillars because taller pillars mean that the velocity gradient in the plastron is smaller, leading to lower viscous dissipation. Thus, the regime where the friction becomes velocity-dependent can be shifted to higher velocities by using taller pillars. As an example, the critical speed beyond which plastron friction dominates is around 10 mm/s for a surface that has a plastron height of 2 μm and a capillary depinning friction of *F*_*s*_ ∼ 10 nN.^70^ According to Backholm et al., the friction in the velocity-dependent regime scales linearly with the capillary number,^70^

9 $$\frac{F}{\gamma_{DA}w}∼\frac{\pi w{\mathrm{Ca}}_{\mathrm{air}}}{4h_{p}}$$

Here, Ca_air_ = η_air_ *V*/*γ*_*DA*_ is the capillary number based on the air viscosity, where *V* is the center-of-mass speed of the drop. The air capillary number is the natural dimensionless number for this problem because the friction is caused by viscous stresses in the air phase. In contrast, Arunachalam et al. proposed scaling law with a different exponent on the capillary number,^71^

10 $$\frac{F}{\gamma_{DA}w}∼40{\mathrm{Ca}}_{\mathrm{air}}^{2/3}$$

While Backholm et al. assumed that the drop remains in contact with the top of the pillars, Arunachalam et al. used interference microscopy to show that an air film is entrained between the top of the solid textures and the drop. The drop lifts off (‘aeroplanes’, in analogy to ‘aquaplane’ when car tires slip on wet roads) and largely loses contact with the surface, as shown by the interferograms in Figure 6e where the interference patterns become smoother as the drop lifts off. When the drop lifts off, details regarding the geometry of the solid and the solid fraction become irrelevant, and the numerical prefactor in eq 10 is thus independent of the solid geometry. To explain the 2/3 exponent on the capillary number in eq 10, Arunachalam et al. used the Landau-Levich-Derjaguin (LLD) framework to calculate friction due to viscous stress in the aeroplaning film.^44^ We fitted the experimental data from both groups between 10^–6^ < Ca_air_ < 10^–4^ and found that the exponent lies between 2/3 and 1 (0.84, as shown by the black line in Figure 7). It is important to note that so far these experiments were performed using the cantilever technique. Reproducing these experiments with different experimental or computational techniques would be valuable to complement these findings because the cantilever can exert an upward capillary force on the drop, which may cause it to lift off prematurely.

A velocity-dependent friction may also arise due to other mechanisms, such as changes in the contact angle hysteresis with velocity, viscous dissipation in the viscous boundary layer inside the drop (Figure 6b), and dissipation in the air wedge surrounding the drop (Figure 6c). However, these sources of friction are believed to be smaller than the friction due to the plastron and air film.^118^

Viscous dissipation due to velocity gradients in the viscous boundary layer inside the drop and dissipation in the air wedge surrounding the drop are estimated to be several times smaller than the dissipation in the plastron and air layer underneath the drop.^118^

At even higher speeds (>10 cm/s), aerodynamic drag becomes important (Figure 6f). By combining experimental data and scaling arguments, Mouterde et al. proposed that the force arising from aerodynamic drag is given by^118^

11 F∼πw24yρairV2Re

where *y* is a numerical prefactor (determined to be 34 in experiments of Mouderde et al.^118^). Re=2ρairRVηair is the Reynolds number of air flowing around the drop of radius *R*. The flow within the drop matters for this regime because the drag coefficient for a rolling object is different from that of a sliding object. To arrive at the conclusion that aerodynamic resistance becomes dominant, Mouterde et al. deposited large drops (100 μL drops) on long surfaces that were inclined by a few degrees (up to around 10°). The experiments required surfaces that were much longer than typical glass slides used in wetting experiments (2.5 m vs 7 cm) because these large drops took around 1 m to reach terminal velocity on inclined superhydrophobic surfaces. The terminal velocity was of the order of several tens of cm/s, which is fast enough for aerodynamic drag to become important. It should be noted that such high velocities are unique to superhydrophobic surfaces. On hydrophobic surfaces, or even liquid-infused surfaces, the velocity of a drop sliding down the surface rarely exceeds a few cm/s without the drop rupturing or forming a rivulet.^127^

When drops with a higher viscosity than water are used (above ∼ 10 mPa s, for example, glycerol or oils), the dissipation due to velocity gradients inside the rolling drop (Figure 6b) dominates the sources of friction described above. Viscous drops of size larger than the capillary length, $\kappa^{-1}=\sqrt{\gamma_{DA}/\rho_{\mathrm{drop}}g}$ are flattened by gravity, leading to the velocity gradients being confined to a height of the order of ∼κ^–1^. The terminal velocity of these large viscous drops rolling down an inclined surface scales as, $V_{0}∼\frac{\gamma_{DA}}{\eta_{\mathrm{drop}}}\;\sin\alpha$ .^118^ Gravity is also important for small viscous drops because the contact radius between the drop and the superhydrophobic surface is still determined by the weight of the drop. For small drops, the drop velocity scales as *V* ∼ *V*_0_κ^–1^/R.^128^ This scaling explains the initially counterintuitive observation that small viscous drops roll down a superhydrophobic surface faster than larger drops. For glycerol drops (η_drop_ ≈ 1 Pa s), the typical drop velocity is of the order of cm/s.^128^

### 3.3 Drop Motion and Friction on Liquid-Infused Surfaces

On a LIS with a fully wetting lubricant that spreads on top of the textures, the drop is not in direct contact with the solid so there is no three-phase contact line between the drop and the solid. While the capillary depinning mechanism that dominates at low speeds on SHS is relevant for LIS with partially wetting lubricants, it is not significant for LIS with fully wetting lubricants. The absence of significant capillary pinning manifests as an exceptionally low static friction.^25^ When the lubricant wets the substrate under the drop, the drop typically begins moving at tilt angles less than 2°, which is about 1–5° smaller than on SHS. Despite the lower static friction on LIS, drops tend to move much slower than on SHS because the kinetic friction on LIS is typically much higher than on SHS. When drops move, the lubricant surrounding the drop gets reorganized. The wetting ridge becomes smaller with increasing speed and it becomes asymmetric, with the front side being shorter than the rear side (Figure 8a,b).^57^ The lubricant layer underneath the drop may also thicken, causing the drop to fully lose contact with the top of the solid and ‘oleoplane’ on a layer of lubricant (Figure 8c,d).^44^ The wetting ridge is a crucial feature that controls the dissipation mechanisms that give rise to friction and how drops transport lubricant as they move on the surface.^51,56^

**Figure 8:**
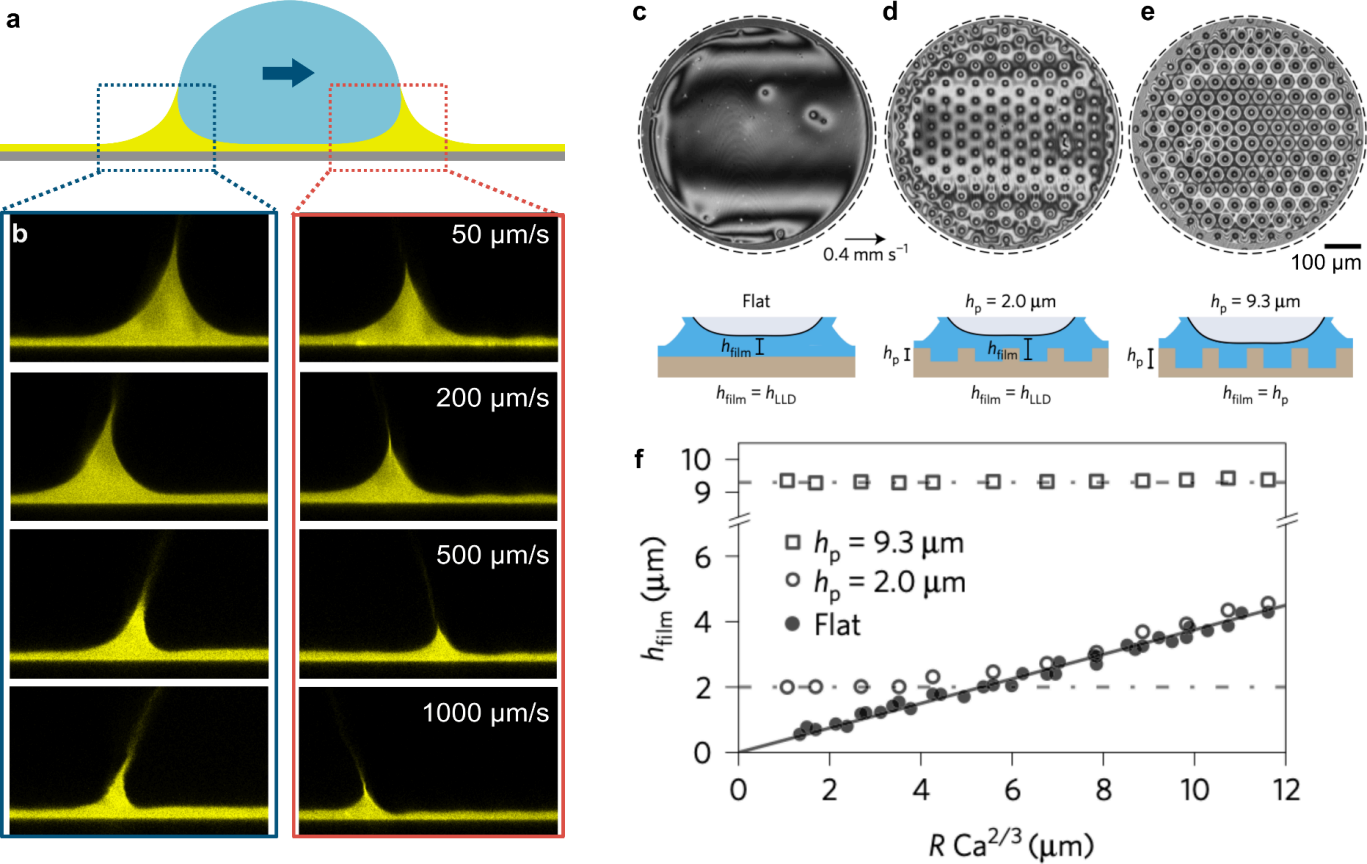
Lubricant reorganization when drops move on lubricated surfaces. (a) Schematic of a drop moving on a LIS with micropillars. The vertical cross section drawn is taken in a slice cutting between two rows of pillars (hence the pillars are not visible in a and b). (b) Shape of the rear (left column) and front (right column) wetting ridge when a water drop moves at different speeds, imaged using an adapted laser scanning confocal microscope setup. The lubricant is 500 cSt silicone oil. The drop is not shown because fluorescent dye was only added to the lubricant. Reproduced with permission under a Creative Commons License CC BY 4.0 from ref 57. Copyright 2024 The Authors. (c–e) Interference patterns obtained when imaging the lubricant film under the drop from below using white-light interferometry. (c) On a hydrophobic flat surface, a lubricant forms under the drop (1 μL); the thickness of the film is given by LLD law. (d) A lubricant film also forms on short pillars (height 2 μm) because the film thickness predicted by LLD law exceeds the pillar height. (e) On tall pillars (9 μm), the LLD height is less than the pillar height and there is no dynamic lubricant film above the pillars, apart from a submicrometer film that remains due to a thermodynamically stable wetting state. (f) The height of the lubricant film under the middle of the drop, measured from the base of the pillars to the drop-lubricant interface (as shown in the schematics of c–e), only increases with Ca (defined in terms of the lubricant viscosity) when the film thickness predicted by the LLD law exceeds the pillar height. These measurements were taken using white-light interferometry. The drop volume was 5–10 μL, the drop speed was 50–600 μm/s, and the lubricant was perfluorinated oil (30–60 cP). Panels c–f are adapted with permission from ref 44. Copyright 2017 SNCSC.

The kinetic friction that drops experience on LIS depends on several factors, including the drop speed, the viscosities of the drop and lubricant, and the geometry of the solid texture.

When the drop viscosity is larger than the lubricant viscosity, energy is predominantly dissipated in the drop due to viscous dissipation arising from velocity gradients in the drop.^51^ In this regime, the velocity of the drop is inversely proportional to its viscosity, similar to what is observed for viscous drops on SHS. The friction scales as, *F* ∼ η_drop_ *VR*.^51^ In this regime, a drop moving on a tilted surface is expected to have a typical velocity of $V∼\frac{\rho_{\mathrm{drop}}gR^{2}}{\eta_{\mathrm{drop}}}\sin\alpha$ , which is of the order of 1 cm/s for a 20 μL drop with viscosity η_drop_ = 1 Pa s.

When the lubricant viscosity is greater than the drop viscosity, friction is predominantly due to viscous dissipation in the lubricant, with dissipation in the drop amounting to less than ≈ 30% of the total energy dissipated.^57^ Figure 9 shows the dependence between the friction force and the capillary number of the lubricant Ca_lub_ = η_lub_ *V*/γ_DL_, where η_lub_ is the lubricant viscosity. Within the lubricant layer, dissipation is distributed predominantly in front and behind the drop within the wetting ridge, particularly at the transition region where the wetting ridge meets the lubricant film underneath the drop (the insets in Figure 9 show heatmaps of where dissipation is localized). Dissipation remains concentrated in the same regions, regardless of whether the surface consists of micropillar arrays or is flat.

**Figure 9:**
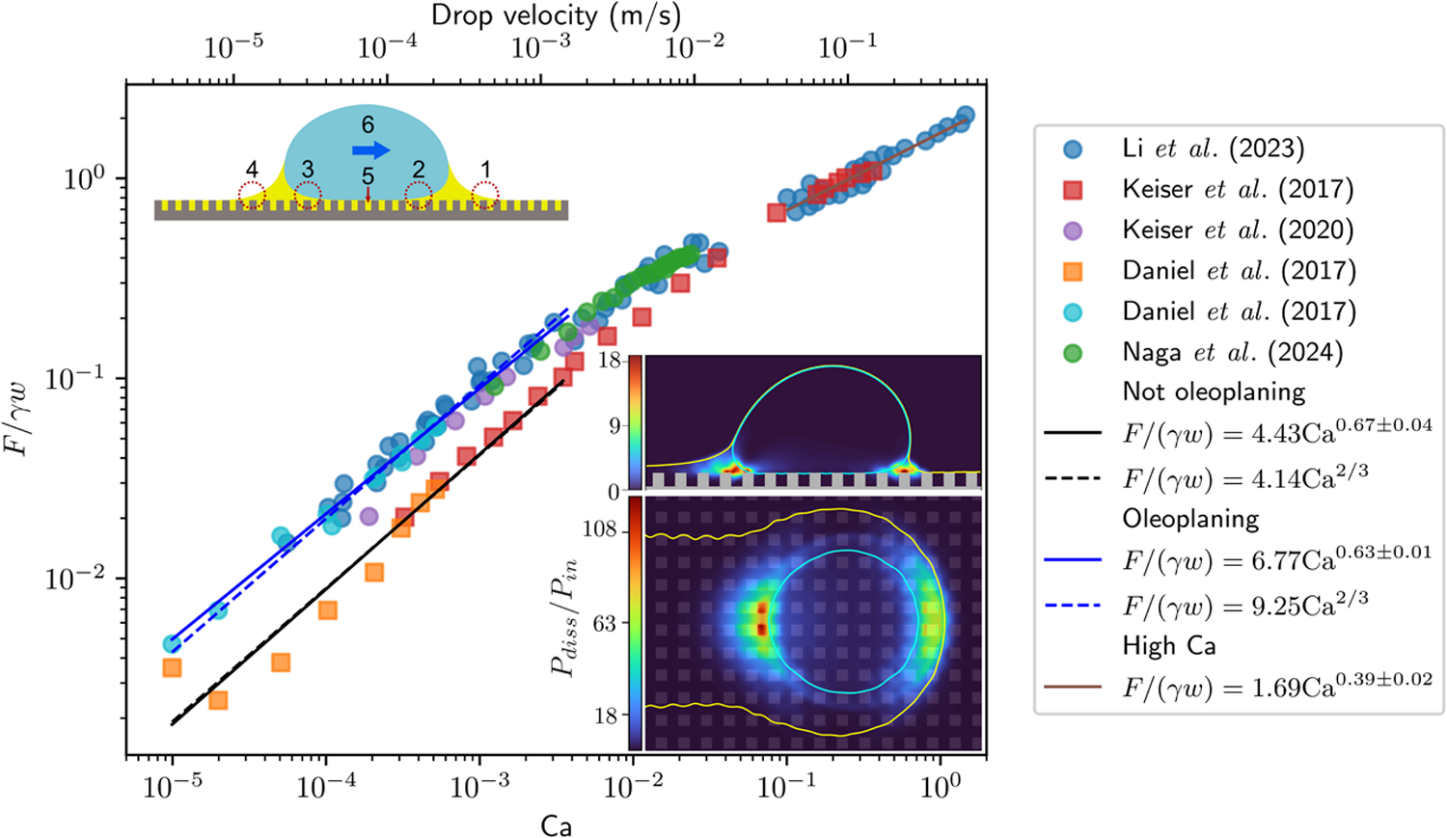
Relationship between friction force and capillary number on lubricated surfaces, where the lubricant viscosity is larger than the drop viscosity. The data are taken from refs 44, 51, 56, 57, and 130 . On the *y*-axis, the friction force is normalized by the product of the drop-lubricant interfacial tension and the drop base width. In cases where the drop base width was not reported in the literature, we estimated it from the drop volume and assumed that the drop is approximately a hemisphere. In the bottom *x*-axis, Ca is defined in terms of the lubricant viscosity and the drop-lubricant interfacial tension. Throughout this figure, γ denotes the drop-lubricant interfacial tension. The drop velocity on the top *x*-axis is computed from Ca, assuming that the lubricant was 100 cSt silicone oil. Note that, since the data points include lubricants of various viscosities, the velocity on the top *x*-axis is not necessarily the true drop velocity but is merely a representative velocity to provide intuition for how fast the drop would move if the lubricant was 100 cSt. Below Ca < 0.005, the data points follow 2 parallel bands. The upper band (circle symbols) corresponds to the case when a Landau–Levich film forms underneath the drop whereas the lower band (square symbols) corresponds to the case where there is no film. For both these regimes, $F/\gamma w∼{\mathrm{Ca}}^{2/3}$ (dashed blue and back line). At Ca ≈ 10^–2^ these 2 bands begin to converge, and we obtain a scaling of *F* ∼ Ca^0.39^ above Ca > 10^–1^ (solid brown line). Solid lines correspond to unbiased fits whereas dashed lines correspond to fits where the gradient is imposed. The top left inset highlights regions where energy may be dissipated in the form of viscous dissipation. Bottom right insets: Heatmaps showing the distribution of viscous dissipation (mainly localized in regions 1,2,3). The term “heatmap” refers to the fact that viscous dissipation is accompanied by release of heat. The top image is a vertical cross section across the center of the drop. The bottom image is a horizontal stack of the dissipation across the entire 3D domain. The drop contour is shown in cyan and the lubricant contour is shown in yellow. The color bars represent the ratio of the dissipated power to the power input per unit volume to move the drop. The top left inset is adapted with permission from ref 56. Copyright 2020 by the American Physical Society. The bottom right insets are reprinted with permission under a Creative Commons License CC BY 4.0 from ref 57. Copyright 2024 The Authors.

Experiments performed in the limit of high lubricant viscosity demonstrate that up to Ca_lub_ ≈ 10^–2^, the friction force scales nonlinearly as,^44,51,56^

12 $$F_{2,4}∼\gamma w{\mathrm{Ca}}_{\mathrm{lub}}^{2/3}$$

Here, the relevant interfacial tension is γ = γ_DL_ for region 2 and γ = γ_LA_ for region 4 (regions labeled in top left inset of Figure 9). The exponent of this scaling law remains 2/3 up to Ca_lub_ ≈ 10^–2^. The scaling law for the friction force (eq 12) typically has a larger prefactor when the drop oleoplanes on a lubricant film (called a Landau–Levich film), but the exponent remains the same, as observed by the two parallel lines (in black and blue) in Figure 9, where the line corresponding to the oleoplaning case (blue line) is higher. The friction arising due to the formation of Landau-Levich films is expected to be localized in the regions where the wetting ridge extends to form a film (regions 2 and 4). The thickness of this lubricant film follows the LLD law, *h* ∼ *w*Ca_lub_^2/3^, as in the case of the air film on SHS, but here the lubricant viscosity enters rather than the air viscosity and the drop-lubricant interfacial tension replaces the drop surface tension. Oloeplaning only occurs when the thickness predicted by the LLD law exceeds the height of the solid textures (Figure 8e,f). The critical lubricant capillary number above which a Landau-Levich film forms underneath the drop scales as, Ca_LL_ ∼ (*h*_*p*_/*R*)^3/2^.^44,129^ Dissipation arising from the formation of Landau-Levich films does not depend on details of the solid texture since these details are obscured by the film.

Friction can also be generated at the leading edge of the front and rear wetting ridges (regions 1 and 3 in the top left inset of Figure 9), as shown in the dissipation heatmaps in Figure 9 (bottom right insets). Unlike the Landau-Levich dissipation mechanism, the friction arising from these regions (1 and 3) depends on the solid fraction because the geometry of the solid texture influences the flow of lubricant locally due to the no-slip boundary condition at the top of the pillars. The leading edge of the wetting ridge slips easily as it moves on the thick oil layer between two pillars but experiences significant dissipation when it moves over the top of the pillars. The friction arising from regions 1 and 3 scales with the solid fraction as, *F*_1/3_ ∼ *γRϕ* ^2/3^ Ca^2/3^, where γ = γ_LA_ for region 1 and γ = γ_DL_ for region 3. This relation between friction and solid fraction was shown to provide good fits to experimental data for solid fractions between ϕ = 23% and ϕ = 52%, but it overestimates the friction for higher solid fractions (ϕ = 67%).

At high capillary numbers (>10^–2^), there is a transition from a 2/3 to an ≈1/3 scaling exponent (brown line in Figure 9).^51,130^ This regime is largely unexplored, and further work is needed to understand the mechanisms at play at these high speeds. It has been proposed that this behavior could be due to the wetting ridge having insufficient time to develop.^130^ Drops may also be dynamically cloaked by lubricant due to the rolling motion of the drop. The cloak can be up to a few micrometres thick when the drop moves fast.^72^ However, current evidence suggests that the cloak does not have a significant influence on the friction.^57^

Bottone et al. observed that when successive drops move along the same trajectory on an initially underfilled LIS, the first drop moves slower than the next drops, implying that the friction decreases with the drop number.^131^ The authors propose that this is due to the formation of a Landau–Levich film at the rear of the drop. This Landau–Levich film gets deposited behind the drop, increasing the lubricant thickness along the trajectory. Consequently, the next drop moves along a trajectory with less underfilling than the previous drop. This result implies that the level of filling affects the friction. Thus, underfilled surfaces have higher friction than perfectly filled and overfilled surfaces.

Vega-Sánchez et al. showed that nanobubbles may form on Teflon wrinkled surfaces infused with lubricant when they are submerged in a bulk flow.^102^ These nanobubbles lead to a significant increase in the measured slip length compared to what is expected on lubricated surfaces.^57^ The nanobubbles effectively create a mixed state between pure SHS and pure LIS. It remains an open question as to whether nanobubbles may also form when drops move on certain types of lubricated surfaces and whether these nanobubbles are relevant to the friction force experienced by drops in certain situations.

### 3.4 Similarities and Differences between Friction on SHS and LIS

Figure 10 summarizes the mechanisms influencing the friction force at different drop speeds; below we elaborate further on the similarities and differences between friction on SHS and LIS.

**Figure 10:**
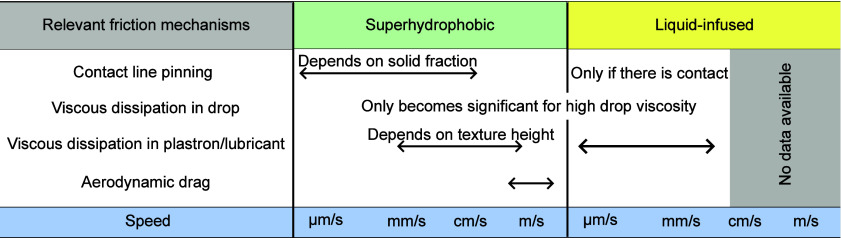
Summary of the mechanisms that contribute to drop friction at different drop speeds on SHS and LIS. Depinning of capillary bridges at the receding side dominates at low speeds. On SHS, depinning can dominate up to speeds of several cm/s when the solid fraction is high (*e.g*., on micropillars). Viscous dissipation in the drop is typically not important for low-viscosity liquids such as water but it becomes important when the drop has high viscosity. As a guideline, the drop viscosity can be considered high when it is above ∼10 mPa s on SHS and above the lubricant viscosity on LIS. On superhydrophobic nanostructured surfaces with low pinning, the drop may lift off at speeds as low as ∼mm/s and air film may form between the drop and the solid textures. The critical speed at which the drop lifts off is higher for taller surface textures. Viscous dissipation in the plastron and air film leads to a velocity-dependent friction force. At speeds of the order of cm/s, aerodynamic drag becomes relevant. On LIS, friction is typically dominated by viscous dissipation in the wetting ridge and where the wetting ridge meets the lubricant film under the drop. On LIS, drops never reach high enough speeds for aerodynamic drag to become important. Current evidence suggests that the presence of a lubricant cloak layer around the drop does not affect the dominant dissipation mechanisms.^57^

#### Similarities

For high drop viscosities, the friction mechanisms are similar on both SHS and LIS; dissipation arises predominantly due to viscous shear in the bulk of the drop. The flow inside the drop follows a mixed rolling/sliding motion, generating velocity gradients which give rise to viscous dissipation. For low drop viscosity (*e.g*., for water), significant differences arise between friction mechanisms on SHS and LIS. However, there is an intermediate speed regime where the friction on these two surfaces has similar origins; this corresponds to the regime where an air or lubricant film forms underneath the drop, causing the drop to lift off and lose contact with the solid. In this regime, the base of the drop and the top of the solid textures are separated by an air or lubricant film. The friction scales with capillary number with a 2/3 exponent (approximately) and there is no dependence on the solid fraction.

#### Differences

The important difference between SHS and LIS is not simply whether the solid structure is filled with air or lubricant, but whether the drop is surrounded by a wetting ridge. This implies that if a drop is placed on a lubricated surface but there is no wetting ridge (*e.g*., by fully submerging the drop and surface in a lubricant bath), the drop dynamics would resemble the dynamics observed on SHS more closely than the dynamics observed on LIS, although the drop velocity would remain much lower than on a SHS. The wetting ridge fundamentally changes the friction mechanisms. Typically, drops have a higher static friction but a lower kinetic friction on SHS. In practice, this means that drops on SHS start moving at a higher tilt angle, but when they move, they travel at much higher speeds than on LIS for the same tilt angle. For low drop viscosities (*e.g*., water), energy is dissipated due to depinning of capillary bridges on SHS, whereas on LIS it is dissipated due to viscous dissipation in the wetting ridge. On SHS the friction is independent of speed at low speeds whereas on LIS, the friction force always has a speed dependence because the dominant dissipation mechanism always has a viscous origin.

In principle, Furmidge’s equation (eq 7) always holds and can be used to estimate the friction force if the apparent advancing and receding contact angles (which become a function of velocity) are known. Typically, the macroscopic contact angles are used. It is not straightforward to obtain the apparent advancing and receding contact angles on SHS since contact angle goniometers are inaccurate for high contact angles although the apparent advancing contact angle is typically close to 180°.^28^ On LIS different contact angles are involved. Daniel et al. argue that the contact angle to enter in Furmidge’s equation the macroscopic contact angle,^25^ which is measured by fitting a circular arc to the drop shape and finding the angle at the point where the circular arc intersects the substrate. In analogy to Amonton’s friction laws between solid objects, Furmidge’s equation can also be written in terms of the normal force between the drop and the substrate,^119^

13 F=μN

where μ = *k*(Θ_*a*_ – Θ_*r*_)/π and *N* = *πwγ* sin Θ_*e*_. Rewriting Furmidge’s equation in this form highlights that to achieve a low friction, we need to not only have a low contact angle hysteresis, but also a small normal adhesion force. The normal adhesion force of a drop on LIS is significantly higher than that on a SHS, typically even exceeding the drop’s weight. From the perspective of eq 13, the static friction on LIS is lower than on SHS despite the higher normal adhesion because the coefficient of friction on LIS is smaller.

## 4 Collapse of the Low Friction States

### 4.1 Collapse of the Superhydrophobic State

The air cushion beneath drops in the superhydrophobic Cassie state may collapse when the shape of the drop–air interface is disturbed, leading to the drop transitioning to a Wenzel state. In the Wenzel state, there is only one interface to consider under the drop: the drop–solid interface. In comparison, in the Cassie state there are two interfaces: the drop–air interface and the solid–air interface. Therefore, the total surface energy is usually lower in the Wenzel state. Although the Wenzel state is a lower energy state, the Cassie to Wenzel transition is not spontaneous because the process involves overcoming an energy barrier. The presence of this energy barrier makes the Cassie state a metastable state. The origins of the energy barrier can be understood intuitively as follows: As the drop sinks into the gaps between the solid structure (*e.g*., pillars), it wets the side walls of the pillars, changing solid–air interfaces to drop–solid interfaces. This process costs energy since drop-solid interfaces are energetically more costly than solid–air interfaces when the equilibrium contact angle is above 90° (eq 1), which is usually the case for water drops on hydrophobic pillars. Therefore, just before the drop touches the bottom substrate and completely displaces the air cushion, the total interfacial energy is greater than that in the initial Cassie state because the energy associated with drop–solid interfaces is increased while solid–air and drop–air interfaces are still present. Mathematically, the energy barrier can be estimated by considering the change in surface energy as the drop sinks into the texture and assuming that the drop/air interface remains flat under the drop,^26^

14 $$\Delta E=\left(\gamma_{SD}-\gamma_{SA}\right)\left(r-1\right)=-\gamma_{DA}\left(r-1\right)\;\cos\Theta_{e}\approx -\frac{2\pi bh_{p}}{p^{2}}\gamma_{DA}\;\cos\Theta_{e}$$

where the last approximation is valid for a solid surface consisting of a regular array of cylindrical pillars of height *h*_p_, radius *b* and pitch (center-to-center) distance *p*. The roughness ratio *r* is defined as the total solid surface area divided by the projected surface area. The Cassie state is metastable only when Θ_*e*_ is greater than the local geometrical angle of the texture, otherwise the transition is spontaneous.^42,132^ The geometric angle is 90° for cylindrical pillars and is higher for re-entrant structures, such as the ones shown in Figure 2d. The energy barrier between the metastable Cassie state and the Wenzel state is much larger than the scale of thermal fluctuations and thus a collapse to the Wenzel state is only possible by supplying energy to the drop, for example by compressing, impacting or vibrating the drop, or due to increase in the Laplace pressure in the drop when it evaporates.

When a drop collapses to the Wenzel state, it becomes strongly pinned. Typically it loses its mobility, which is why the Wenzel state is undesirable. Additionally, its apparent contact angle falls to a lower value, given by the Wenzel equation in thermodynamic equilibrium,^37^

15 $$\cos\Theta_{w}^{\mathrm{app}}=r\;\cos\Theta_{e}$$

This equation is obtained by setting d*E*_*W*_ = 0 in eq 3 since the change interfacial energy should be zero in thermodynamic equilibrium. The Wenzel angle is always smaller than the Cassie–Baxter angle. Again, the Wenzel angle does not account for contact line pinning. Contact line pinning is even more severe than for drops in the Cassie state, often exceeding 50°. Due to the large contact angle hysteresis, the measured contact angles differ significantly from the thermodynamic value predicted by eq 15.

Early studies compared the stability of the Cassie and Wenzel states based solely on the respective total surface energies.^133^ They concluded that the metastable Cassie state on SHS becomes increasingly favorable with taller hydrophobic protrusions because taller protrusions have a larger Cassie to Wenzel energy barrier due to the greater surface area that needs to be wetted on the side walls of the protrusions. This effect is captured in eq 14, where the energy barrier is proportional to the pillar height. However, this comparison is only valid under zero pressure, for which the assumption of a straight (no curvature) drop-air interface under the drop holds. Pressure induces curvature of the drop-air interface (Figure 11a),^134^ changing the energy barrier between the Cassie and Wenzel states.^135,136^ The pressure threshold for the collapse of the Cassie state has been calculated using a force balance on pillars or by calculating the total energy.^137,138^ The drop curvature is assumed to be the same everywhere, ignoring the effects of gravity. Thus, the drop-air interface between pillars is more curved for small drops and “sags” to a maximum depth δ that scales as δ ∼ *p* ^2^/*R*, where *R* is the radius of the spherical cap of the drop. This is a rough approximation that is strictly valid only for one-dimensional channels. Pressure can be applied in different ways, such as changing the size of the drop,^73,139^ impact,^140^ vibrations, or squeezing the drop.^52^ Alternatively, curvature can change through electrowetting.^141^

**Figure 11:**
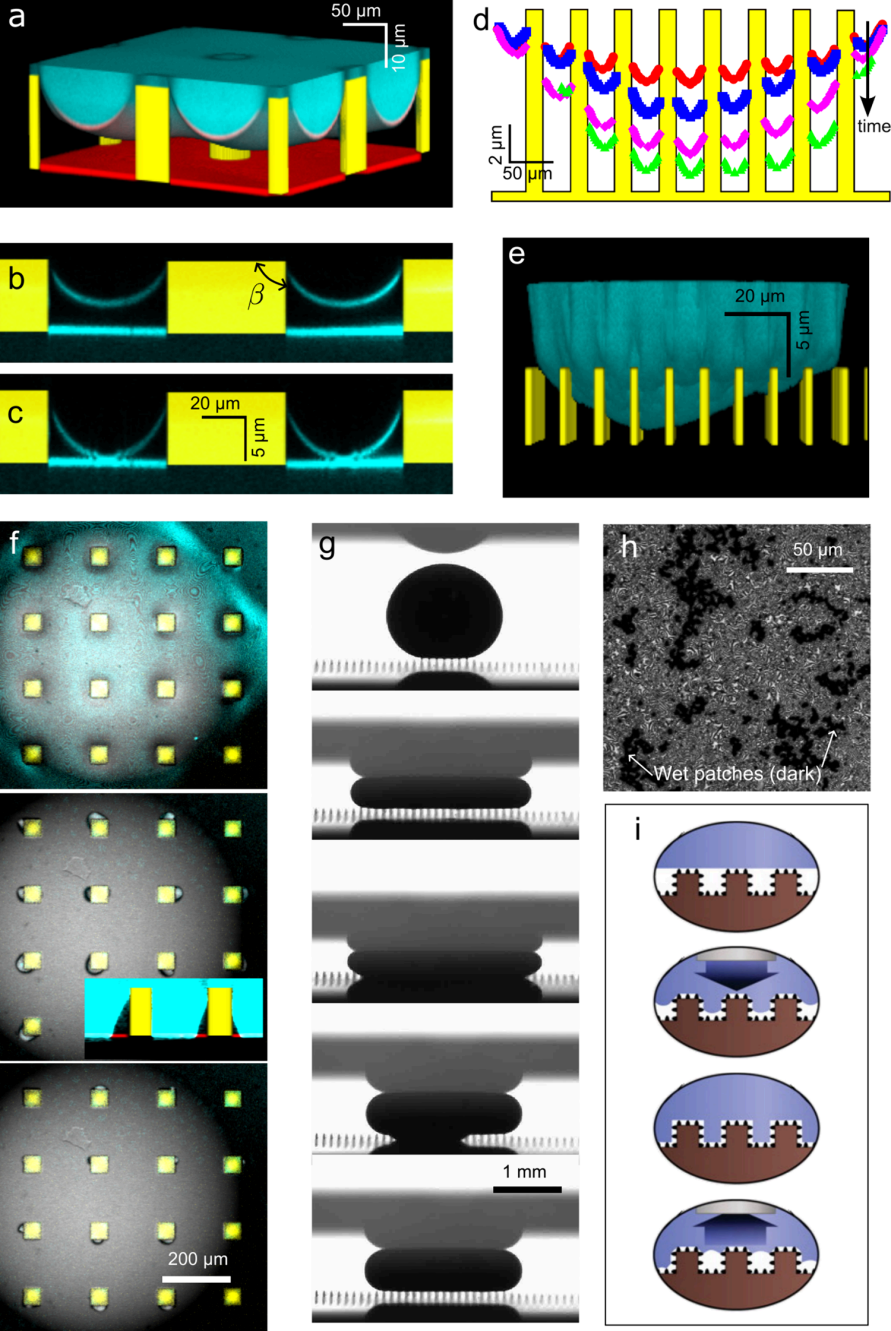
Collapse of the Cassie state on superhydrophobic surfaces. (a) 3D confocal microscopy image (water fluorescence in cyan, reflection from the drop-air interface in red) of the center of a water drop sitting on hydrophobized micropillars (yellow), showing the curvature of the interface. The vertical axis is scaled differently to the horizontal axis to highlight the curved drop-air interface. Adapted from ref 160. (b, c) Sagging mechanism. Confocal microscopy vertical slice through the deepest point of the drop-air interface (cyan). Laplace pressure increases as the drop evaporates and the interface eventually touches the substrate while the microscopic contact line remains pinned at the edges of the pillars. Adapted with permission from ref 47 under a standard PNAS License. (d) Depinning mechanism. The microscopic contact line slides down the side of the pillars when the contact angle with respect to the vertical reaches the material’s contact angle. These metastable intermediate wetting states can last for several seconds. Adapted with permission from ref 48. Copyright 2007 by the SNCSC. (e) 3D confocal image of an intermediate wetting state. Adapted with permission from ref 47 under a standard PNAS License. (f) Composite confocal and transmission images of an evaporating water drop sitting on micropillars. Just before the transition via the sagging mechanism, interference fringes are visible. When the air cushion vanishes, they disappear. Air bubbles get trapped around the micropillars, shrinking slowly as air diffuses into the water drop. The inset shows a vertical slice of bubbles (black) trapped on the side walls of pillars (yellow) under fluorescently labeled water, imaged with confocal microscopy. Adapted with permission from ref 47, under a standard PNAS License. (g) Reversibility of the Cassie state was experimentally confirmed on micropillars with high aspect ratio coated with hydrophobic nanoparticles. Here pressure is applied directly by squeezing the drop between two plates. Adapted from ref 153, PNAS Open Access. (h) Reflection interference from underneath a water drop sitting on a disordered structure of hydrophobic raspberry-like particles. Wenzel islands (dark patches) that grow with time may appear. Unpublished data. (i) On dual scale structures, featuring nanoprotrusions on top of micropillars, the Cassie-to-Wenzel transition may be reversible because a thin air cushion remains trapped, causing the drop to remain in a nano-Cassie state despite being in a micro-Wenzel state. Adapted from ref 157, PNAS Open Access.

It was predicted theoretically and verified experimentally by various microscopy techniques that the collapse of the Cassie state proceeds through two basic mechanisms: “sagging” and “depinning”.^75^ These mechanisms have been given a variety of names, but the various definitions can be grouped into these two categories.^74^ In the sagging mechanism, the contact line remains pinned at the top edges of the solid textures and the drop curvature increases to the point that it touches the substrate between the protrusions (Figure 11b,c). A new contact line with an unstable instantaneous contact angle of 180° forms, forcing the liquid to spread fast through the protrusions, with a speed up to approximately 10 m/s.^142^ On model micropillar substrates the deepest point is near the center of the drop and the collapse is triggered as soon as the drop touches the bottom substrate at any single point. An order of magnitude estimate for the critical drop radius below which the drop collapses to the Wenzel state due to sagging can be obtained by equating the curvature of the drop-air interface to the texture height. On pillars, the critical radius scales as^26,73^

16 $$R^{*}∼\frac{p^{2}}{h_{p}}$$

The sagging collapse is more common for protrusions of low aspect ratio (smaller *h*/*p*) because, as highlighted by eq 16, a smaller curvature (larger drop radius) is required for the liquid to contact the base of the substrate when the aspect ratio is smaller.

The depinning mechanism occurs when the increasing curvature under the drop forces the drop-air interface to form an increasing angle (measured in the drop phase) with respect to the dry solid and eventually depin from the top edges of the pillars. When the material contact angle exceeds the advancing contact angle, the contact line starts to slide along the previously dry solid. The critical contact angle is given by the Gibbs’ criterion, Θ_Crit_ = (π – β) + Θ_e_, where β is the local geometrical angle of the edge (Figure 11b).^143^ The pressure limit at which depinning collapse occurs depends on the spacing and shape of the protrusions, especially whether the shape has re-entrant structures.^42,58,144–146^

Zheng et al. gave a generic formula for this pressure limit for pillars with an arbitrary cross-section,^137^

17 $$P_{\mathrm{Crit}}=-\frac{\gamma_{\mathrm{DA}}f\;\cos\Theta_{\mathrm{Crit}}}{\left(1-f\right)\lambda }$$

where λ is defined as the ratio of cross-section area to the perimeter of the pillars. In contrast to the sagging mechanism, this limit is independent of the height of the protrusions. Therefore, the collapse of the Cassie state can happen on any structure. The depinning collapse is a coordinated process where the exact shape of the drop-air interface locally between 2 protrusions is affected by depinning on neighboring protrusions. Depinning collapse is not as abrupt as sagging and may take several seconds to complete on model micropillar arrays, even at constant pressure (Figure 11d,e). Both the sagging and depinning mechanisms can be inhibited by employing structures that are as small as possible.^59,76,147^ However, simulations and experiments on nanostructures show that the critical pressure may be lower than the one predicted by macroscopic models.^148^

During collapse, the curved drop-air interface underneath the drop does not always displace the air cushion completely. On irregular structures, the collapse may proceed via either the sagging or depinning mechanism, depending on local variations of substrate geometry, and is a quasi-stochastic process. The Cassie and Wenzel states may coexist, as shown in Figure 11h where the dark patches correspond to regions where the drop has collapsed and the lighter background corresponds to regions where the drop is still in the Cassie state. Even on regular micropillar substrates some air bubbles remain trapped around the walls of the micropillars (Figure 11f), especially when the collapse of the Cassie state proceeds via the sagging mechanism. But these trapped air bubbles are unstable because the Laplace pressure within the bubbles is higher than the outside pressure, causing the air to diffuse into the liquid.^149^ Depending on the initial size of the bubbles, the exact geometry of the substrate, and the degree of saturation of the liquid, bubble lifetimes may range from milliseconds to several hours or even days.^102,150,151^ But ultimately, the liquid reaches the fully wet Wenzel state.

In some cases, it is possible to recover from a Wenzel state to a Cassie state by releasing the applied pressure or inputting energy into the system, for example by applying vibrations.^152^ There is even a regime where the Cassie state is the only energy minimum and is preferred over the Wenzel state. The criterion that needs to be fulfilled to obtain such a monostable Cassie state is,^153^

18 $$\frac{1-f}{r-f}<-\cos\Theta_{r}$$

Here, the local receding contact angle, Θ_*r*_ is the relevant contact angle because this condition is derived by considering the dewetting process during a Wenzel to Cassie transition. During dewetting, the drop-air interface recedes. For a water drop on hydrophobic micropillars, with a material receding contact angle of 100° (on flat surfaces), the criterion for a monostable Cassie state is only satisfied when the pillars have an impractically large aspect ratio, *h*_*p*_/*b* > 11 (assuming the solid fraction is *f* = 0.1). It is difficult to fabricate pillars with such high aspect ratios and even if they can be fabricated, they will be mechanically very fragile. The criterion to obtain a monostable Cassie state can be made more lenient by coating the micropillars with nanostructures to induce a nano-Cassie state that increases Θ_*r*_ to around 150°. This high receding angle reduces the required aspect ratio to *h*_*p*_/*b* > 0.4. Although the Wenzel-to-Cassie transition is theoretically possible, it has been experimentally observed and discussed only in a limited number of studies. Li et al. applied pressure to sessile drops on micropillars by squeezing them with a hydrophobic plate (Figure 11g).^153^ The drop transitioned to the Wenzel state through the depinning mechanism. When the pressure was released, in some cases, the drop went back to the Cassie state when the inequality in eq 18 was fulfilled. The mechanism of the Wenzel-to-Cassie transition resembled an ‘unzipping’ process, where the drop edges unzip first and the unzipping front propagated toward the middle of the drop (Figure 11g).

Condensation can sometimes cause drops to transit from a Wenzel state to a Cassie state even when the Cassie state is not monostable. In a study where the size of Wenzel drops increased through condensation, some but not all drops transitioned to the Cassie state.^154^ The mixed outcome was attributed to local pinning effects. The transition to the Cassie state is favored by the coalescence of adjacent drops. During coalescence, drop-air surface area reduces, releasing interfacial energy that can overcome the Wenzel-to-Cassie energy barrier. Another study found that condensate drops may only be in a partially wetted Wenzel state, reducing the barrier for the transition to the Cassie state.^155^ If a thin air cushion remains after the collapse,^156^ such as on structures that combine micro- and nanometer length scales,^157^ the reversal is relatively easy because the drop remains in a nano-Cassie state and never fully collapses to the Wenzel state (Figure 11i). However, the small amount of air in the nano-Cassie plastron may gradually diffuse into the liquid, which raises the question of its longevity. The effect of prolonged stay in the Wenzel state on the success of the Wenzel-to-Cassie transition has not been studied in detail.

It is also possible to lift a drop from the Wenzel state by adding lubricant to the substrate, provided that the lubricant is immiscible with the drop and wets the solid even under the drop.^158,159^ This essentially converts an SHS to LIS.

### 4.2 Collapse of the Lubricated State

Depletion and dewetting of the lubricant can induce the collapse of the lubricated Cassie state on LIS. Depletion can be caused by leakage as lubricant spreads onto contacting objects, gravitational drainage, and removal by the wetting ridge and cloak when drops slide off the surface. Depletion has two negative consequences. First, it leads to a degradation in the slippery performance of LIS because a reduction in the amount of lubricant can expose the underlying solid structure, leading to enhancing pinning sites for subsequent drops.

Second, any depleted lubricant is transferred from the LIS to either the drop or the object onto which the lubricant has leaked. Thus, the depleted lubricant effectively pollutes the object, which can be messy and is often undesired in industries such as the food, health, and cosmetic industries.

The arrangement of lubricant after partial depletion by a bulk flow of water (i.e., no wetting ridge) was observed on the nanoscale by mapping the lubricant thickness using atomic force microscopy.^161^ The contour maps of lubricant distribution revealed the position of the lubricant contact line on the topography of the substrate (Teflon wrinkles). Upon lubricant depletion, deeper regions of the nanostructure remained covered in lubricant while shallower regions became depleted and exposed to water. These contour maps were used to quantify the volume of lubricant required for effective antifouling behavior, which was as low as 0.2 mL/m^2^.^16^ However, no wetting ridge was present in these experiments and dynamic information on the mechanism of collapse could not be obtained using atomic force microscopy.

When a wetting ridge is present (i.e., with drops), local depletion of lubricant caused by the formation of the wetting ridge plays a crucial role in determining the stability of the lubricated Cassie state. When the wetting ridge forms, it draws lubricant from its surroundings and from underneath the drop.^96^ During this process, lubricant gets depleted at the edges of the drop, which may trigger a collapse from the lubricated Cassie to the Wenzel state at the drop edge.

Scaratt used confocal microscopy to image how drops transit from a lubricated Cassie state to a partial or complete Wenzel state on silicone oil-infused micropillared surfaces that were either hydrophilic (Figure 12a) or hydrophobic (coated with PDMS brushes, Figure 12b).^162^ In these experiments, both the hydrophilic and hydrophobic pillars correspond to the regime where the lubricant does not completely wet the top of the pillars under the drop (i.e., the spreading parameter of lubricant under the drop is negative) and forms a nonzero contact angle on the pillars.

**Figure 12:**
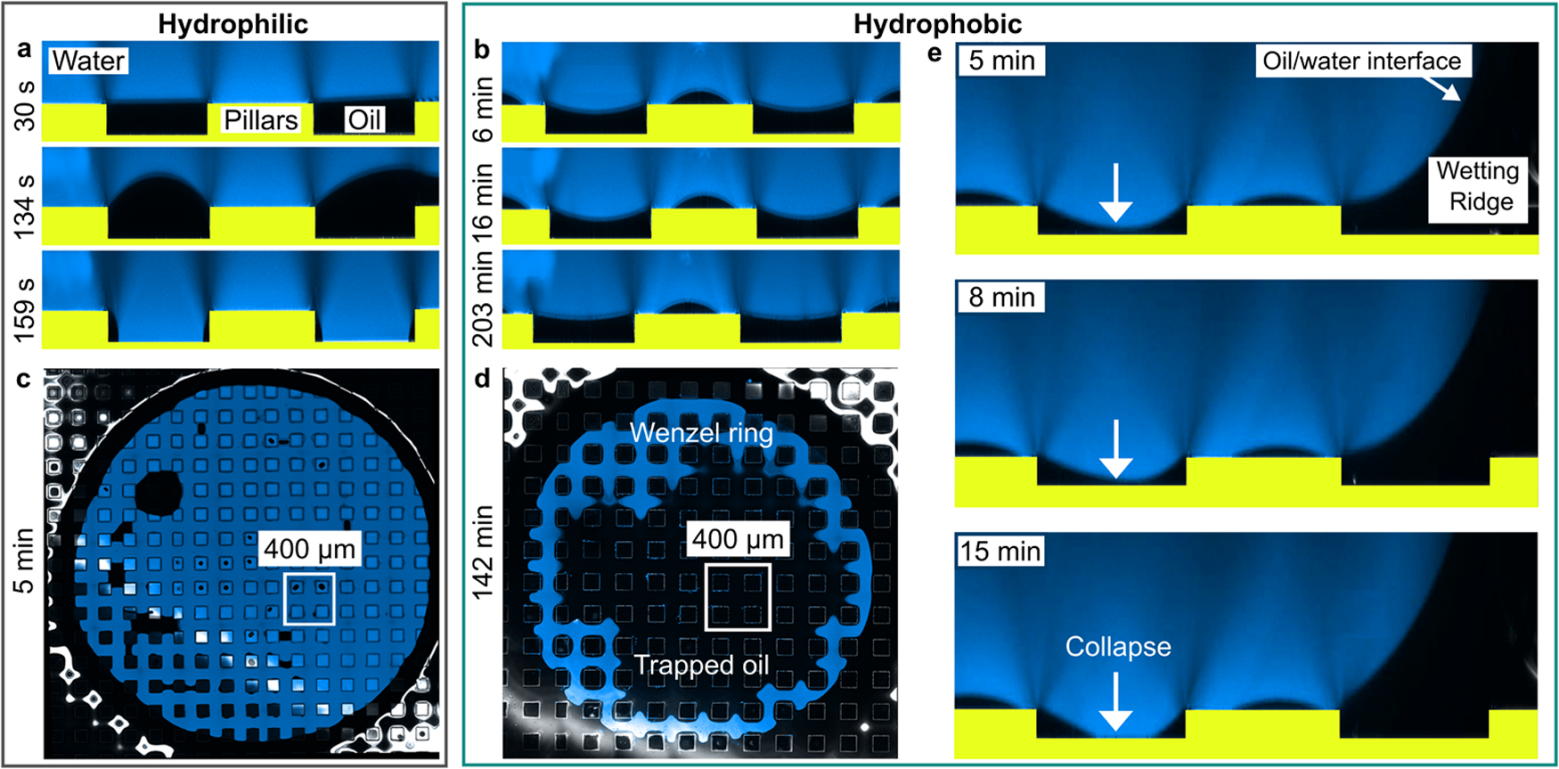
Confocal microscopy images showing the collapse of the lubricated Cassie state on LIS. The lubricant (black) is silicone oil (10,000 cSt), the micropillars (yellow) have a height of 10 μm and width of 100 μm. The drop is water (5 μL, blue). Initially, the lubricant height is equal to the pillar height (perfectly filled). (a) On hydrophilic (untreated SU-8) pillars, the drop displaces the lubricant between the pillars and collapses to the Wenzel state within 159 s. (b) On hydrophobic (PDMS brush-coated) pillars, lubricant remains trapped between the pillars and the middle of the drop remains in a Cassie state even after 203 min. The images in (a) and (b) show a vertical confocal slice taken close to the middle of the drop. (c) Horizontal confocal slice taken at the top of the hydrophilic pillars shows that only a few isolated lubricant pockets remain (dark patches within the blue region) due to pinning after the drop has collapsed between the hydrophilic pillars. (d) Horizontal slice taken at the base of the hydrophobic pillars shows that a large pocket of lubricant remains trapped in the middle of the drop inside a Wenzel ring. The lubricant in this pocket remains trapped until the end of the measurement because it is blocked by the Wenzel ring. White boxes in (c) and (d) highlight regions used for vertical cross sections in (a) and (b), respectively. (e) Vertical slice of collapse dynamics at the edge of the drop on hydrophobic pillars. The snapshots show how the drop-lubricant sags to collapse to the Wenzel state at the drop edge. This collapse occurs around the circular drop contact perimeter, leading to the Wenzel ring shown in (d). Note that the aspect ratio of the images is not 1 in the vertical slices (a,b,e). In these panels, the vertical axis has been magnified to highlight the curvature of the drop-lubricant interface and the collapse. Adapted from ref 162, with permission from the author.

On the lubricated hydrophilic pillars, the drop displaced the lubricant and collapsed to a Wenzel state within a few minutes (for a lubricant viscosity of 10,000 cSt), Figure 12a. The point where the collapse initiates appears to be random. At the onset of collapse, reorganization of lubricant can locally lead to an increase of the thickness of the lubricant film (Figure 12a, 134 s). Once the collapse is initiated, the collapsed front quickly propagates outward, causing neighboring regions to collapse as well. Small localized pockets of lubricant may remain trapped underneath the drop, likely due to pinning at surface imperfections or due to being suddenly isolated from displaced lubricant (black regions in the top left region of the blue area in Figure 12c). Collapse also occurred when the pillars were initially overfilled with lubricant. Overfilling slows down the collapse but does not prevent it.

On hydrophobic pillars, the collapse of the lubricated Cassie state was much slower and the final state was also different. A large pocket of lubricant remained under the middle of the drop. The lubricant pocket remained stable until the end of the measurement (over 2 h) (Figure 12b). The lubricant pocket remained trapped because the outer perimeter of the drop collapsed to the Wenzel state, forming a closed ring that prevents lubricant within the ring to flow outward (the blue ring in Figure 12d shows the collapsed ring). The formation and growth of the wetting ridge drives the Wenzel collapse at the perimeter. While the collapse is a transient process that depends on the rate of growth of the wetting ridge, the final state itself is a metastable state with a Wenzel ring at the perimeter and a lubricated Cassie state in the middle. Thus, the wetting ridge can fundamentally change the properties and appearance of the metastable state on LIS compared to SHS.

Side view images (Figure 12e) reveal that the collapse occurred via a sagging mechanism. The outcome remained unchanged when the pillars were initially overfilled, but the collapse took longer. The energy barrier for this sagging collapse can be estimated using eq 14, substituting the surface tension with the drop-lubricant interfacial tension and inserting the material contact angle between the drop-lubricant interface and the pillar. As is the case for superhydrophobic surfaces, the Cassie state can be made more resistant to sagging collapse by increasing the magnitude of the energy barrier. This can be achieved by increasing the pillar height and width, or decreasing the pitch distance.

The collapse from a lubricated Cassie state to a partial or complete Wenzel state could also be inferred by measuring the tilt angle at which the drop rolled off the LIS. When the LIS was tilted shortly after depositing a drop, the drop typically rolled off at an inclination of less than 5°, regardless of the initial lubricant thickness. Low roll-off angles could also be obtained on overfilled hydrophilic pillars with excess viscous lubricant as long as the tilt angle was measured before the Cassie state collapsed. However, when the drop was left on the surface for an extended time (*e.g*., 30 min), the roll-off angle increased significantly, indicating that the drop had partly or completely collapsed to the Wenzel state. In general, several factors can contribute to the collapse on LIS, including the dewetting of lubricant by the drop, the growth of the wetting ridge, and an increase in Laplace pressure as the drop evaporates.^163,164^ We note that in some cases, the roll-off angle may not increase significantly after the collapse of the micro-Wenzel state if a nano-Cassie state or a nanoscopic lubricant film, stabilized by disjoining pressure, remains (Figure 3d).^95^

### 4.3 Similarities and Differences between SHS and LIS Transition Behavior

#### Similarities

Drops on both SHS and LIS are prone to collapsing irreversibly from the Cassie state to a partial or complete Wenzel state when sufficient pressure is applied because the Wenzel state is usually more energetically favorable. In both cases, the surface chemistry of the solid texture plays a crucial role in determining the critical pressure that needs to be exceeded for the drop to collapse. On LIS, surface chemistry matters even when the top of the solid texture is initially completely covered by a layer of lubricant.^72,165^ To achieve high stability of the Cassie state, the solid must be hydrophobic such that the material advancing contact angle between the drop and the solid is as high as possible. On LIS, the relevant contact angle that determines the stability of the Cassie state is the apparent advancing contact angle between the drop-lubricant interface and the solid. This angle lies within the wetting ridge and can only be resolved using fluorescence or confocal microscopy. Both types of surfaces can undergo collapse via the sagging or depinning mechanism. The sagging mechanism can be suppressed by using tall pillars such that the curved interface of the drop between adjacent pillars does not touch the bottom of the solid substrate. For both surfaces, re-entrant structures can be used to enhance the stability of the Cassie state by making it harder to achieve the criterion for a depinning collapse.

#### Differences

On SHS the Cassie state usually collapses via the depinning mechanism. On hydrophobic structures with a regular pattern (*e.g*., pillar arrays), the collapse typically starts at the center of the drop on SHS. In contrast, on LIS the collapse tends to start at the edge of the drop due to the growth of the wetting ridge, causing lubricant depletion at the edge, which drives the formation of a Wenzel ring at the perimeter. On SHS, any defects in the solid texture (*e.g*., a broken pillar or a patch that has not been properly hydrophobized) will act as a favorable spot to trigger the collapse. Thus, a superhydrophobic surface is only as strong as its weakest point. Defects in the solid texture are less critical on LIS because the lubricant tends to reconfigure and spread under the droplet, smoothing out imperfections. When the surface texture is stochastic, the collapse on SHS is no longer initiated in predictable locations because it depends on the local variations in the texture geometry.

Due to the positively curved drop interface, pockets of air or lubricant get trapped at the bottom of the pillars when a drop collapses on SHS or LIS, respectively. This is more evident with the sagging mechanism. On SHS, the trapped air eventually diffuses into the drop, causing the texture to be fully invaded by liquid. But on LIS, the lubricant remains as the lubricant does not dissolve in the drop and barely diffuses on the time scale of typical experiments. On irregular structures such as Teflon wrinkles, the bottom of the texture is often smaller (nanoscale) than the tops, leading to a higher interfacial curvature of the lubricant and therefore a progressively higher retaining force as the lubricant depletes.

On SHS, the Cassie state is typically metastable. However, on LIS the lubricated Cassie state is not initially metastable even when the material contact angle of the drop on the solid in the presence of lubricant is as high as 140° (Figure 12e), which is higher than the material contact angle on SHS (limited to 120° for water when the surrounding phase is air).

The growth of the wetting ridge depletes lubricant at the edge of the drop, causing the lubricated Cassie state to transit to a state where only the outer drop perimeter collapses to form a Wenzel ring with one or several lubricant pockets trapped inside. This state is metastable because lubricant that is trapped within the collapsed ring can no longer rearrange as there is no pathway for it to flow outward. Thus, in this case, the metastable state is not a fully lubricated Cassie state, but one where the drop only remains in a lubricated Cassie state in the middle but collapses to the Wenzel state at the perimeter.

For certain combinations of drop and lubricant, repulsive disjoining pressures can prevent direct contact between the drop and the solid by stabilizing a thin lubricant film between the drop and the solid. This thin lubricant film will lead to a lower friction than if full contact had been made and it may help recover back to the Cassie state more easily. On SHS, the disjoining pressure in the air film present just before the drop wets the solid substrate is usually attractive, favoring film rupture.

## 5 Perspective and Open Questions

In this section, we highlight some fundamental questions that we believe remain unsolved. We hope that these will inspire the community to advance these topics.

1. *Determining scaling law prefactors for geometries that appear similar when fine details are ignored*. Scaling laws have been highly successful in describing trends and identifying relevant physical mechanisms to explain the origins of friction. However, prefactors that enter in these scaling laws can differ significantly for different geometries and remain to be determined systematically. For example, scaling laws would typically predict that two surface textures with the same solid fraction but different shapes have similar friction characteristics. However, this might not be the case because geometries with sharp edges will pin contact lines more strongly than geometries with smooth edges. With new 3D printing technologies, such as two-photon polymerization, it is becoming possible to fabricate regular and random structures with nanoscale precision. Future advancements will provide the opportunity to experimentally explore the influence of edges and hierarchical roughness in a systematic way.
2. *Is the Cassie state more stable on liquid-infused surfaces?* One of the motivations behind the design of liquid-infused surfaces is that it has a greater resistance to collapse of the Cassie state under increased pressure (*e.g*., due to drop evaporation). To our knowledge, the stability of the Cassie state of drops has not been directly compared on superhydrophobic and liquid-infused surfaces as drops evaporate. It is not trivial to predict which surface will collapse to a Wenzel state first when a drop evaporates, even when we assume that both surfaces have the same geometry and the drop volume is the same initially. Various factors need to be considered, including how fast the drop evaporates on each surface, the rate of growth of the wetting ridge, and whether the collapse proceeds via a sagging or a depinning mechanism.
3. *Are overhangs useful for liquid-infused surfaces?* It is known that structures with overhangs increase the stability of the Cassie state on superhydrophobic surfaces because they pin the contact line more strongly underneath the drop, thus prolonging the onset of collapse via depinning. On liquid-infused surfaces, our preliminary data on pillars shows that the drop/lubricant interface under the drop remains pinned at the edges of the pillars and the collapse to a Wenzel state proceeds via a sagging mechanism. Since overhangs do not inhibit the sagging more than structures with no overhangs, it is not clear whether overhangs must be considered when designing liquid-infused surfaces. Overhangs are expected to minimize lubricant depletion, but they could also be more difficult to imbibe from the top if they are oil-repellent.
4. *Are there optimal geometries that minimize friction while maximizing stability?* Friction and stability of the Cassie state tends to be studied separately. However, an optimal surface design must have both a low friction and at the same time be robust to Wenzel collapse when pressure is applied or as drops evaporate. Structures that have low drop friction do not necessarily have high stability of the Cassie state. For example, sparse pillars with low solid fraction may have very low friction due to the small contact area, but they would easily transition to the Wenzel state. Multivariate optimization could be used to select optimal geometries for specific applications by defining cost functions that account for both friction and stability of the Cassie state.
5. *Are the collapsed states equally bad on both surfaces?* On superhydrophobic surfaces, water quickly wets part of/the entire surface textures once the liquid front touches the base of the substrate at any given point. On lubricated surfaces, when the edge of the drop collapses, lubricant remains trapped in the middle. The drop rests in a mixed Cassie–Wenzel state. The trapped lubricant cannot dissolve into the drop, as is the case for trapped air pockets after collapse on a superhydrophobic surface. If the edges typically collapse first on liquid-infused surfaces, the drop sits in a state that is partly Wenzel and partly Cassie. It would be interesting to compare the pinning forces that oppose movement of a collapsed drop on superhydrophobic and liquid-infused surfaces and explore whether one type of collapse is more catastrophic than the other in the context of drop mobility. Another related open question is whether a small drop with a larger curvature (more sagging between pillars) underneath the drop has different dynamics than a large drop with a relatively flat interface.
6. *Are there similarities between the dynamics of a drop aeroplaning on a SHS and a drop in a Leidenfrost state?* One might expect the friction of an aeroplaning drop to be similar to the friction of a drop moving in a Leidenfrost state since in both cases the drop is not in contact with the surface. However, there is a key difference between these two situations: the equilibrium thickness of the air film underneath a static drop is of the order of microns,^167^ whereas on SHS, there is no air film between the top of the protrusions and the drop in equilibrium). When the drop is set into motion, the film thickness will increase as a function of velocity only above a critical speed beyond which the thickness predicted by the LLD law exceeds the static thickness. Due to the larger equilibrium thickness of the Leidenfrost state, we expect the critical velocity above which the film thickness begins to follow the LLD law to be significantly higher for the Leidenfrost state.
7. *Influence of nanobubbles*. When a hydrophobic solid structure of LIS is exposed directly to water under microfluidic flow, nanobubbles can nucleate or remain entrained.^166^ Even if the surface coverage of gas on LIS remaines low (lower than 20% of the surface in the case of immersed Teflon wrinkles), it is found to be sufficient to induce large microscale slip under continuous microfluidic flow. Bubble nucleation could be related to the level of lubricant depletion: bubbles nucleate when the highly hydrophobic structure comes in direct contact with water, and once formed, the bubbles further displace the lubricant away from them. LIS are more stable to applied static pressure than SHS under microfluidic flow.^166^ The measured slip length on LIS did not decrease much when the pressure was increased from 0 to 200 kPa, while the slip length on SHS decreased dramatically as soon as 50 kPa of static pressure was applied. The decrease in slip length is interpreted as a collapse from a Cassie to a Wenzel state. This suggests that the Cassie state remains stable under greater pressures on LIS, despite the presence of bubbles on them. However, lubricant behavior in microfluidic flow is different to that under a drop due to the absence of the wetting ridge in bulk microfluidic flow. As discussed above, the growth of the wetting ridge affects the stability of the Cassie state. Further work is required to understand whether nanobubbles are relevant for drops and whether the Wenzel collapse in a microfluidic channel follows the same mechanism as that of a drop.

## Vocabulary

Superhydrophobic surface (SHS): A solid surface with chemical hydrophobicity and physical roughness on the microscale and/or nanoscale. Water drops are very mobile on SHS. A natural example of SHS is the lotus leaf.
Liquid-infused surface (LIS): A solid surface with roughness on the microscale and/or nanoscale that is imbibed with a liquid lubricant. The solid and lubricant must be chemically compatible, which means that the contact angle the lubricant forms with the solid surface has to be lower than the critical contact angle required for the lubricant to spontaneously wick the solid structure. The lubricant and drop must be immiscible. Drops are mobile on LIS but typically move much slower than on SHS.
Drop friction: The force that opposes the motion of liquid drops when they slide or roll on a solid surface. Drop friction can be divided into static and kinetic friction. Static drop friction is the force that needs to be overcome to initiate drop motion. Kinetic drop friction is the force that the drop experiences when moving on a surface. LIS typically has a lower static drop friction but a higher kinetic drop friction than SHS. In general, drop friction may arise due to different factors, including due to surface defects, viscous dissipation, electrostatic retardation, surface adaptation, and aerodynamic resistance.
Wetting ridge: The annular lubricant region that rises around a drop when the drop is placed on LIS. The wetting ridge controls drop friction on LIS when the lubricant is more viscous than the drop.
Cassie state: The configuration where drops rest on top of the solid roughness when placed on SHS/LIS. Air (lubricant) pockets remain in the gaps between the rough structures on SHS (LIS). The drop only makes partial contact with the solid.
Wenzel state: The configuration where drops displace the air (lubricant) pockets under the drop and invade the gaps between the rough solid structures on SHS (LIS). The Wenzel state typically has a higher drop friction than the Cassie state. Drops can collapse from the Cassie to the Wenzel state due to an increase in pressure (*e.g.*, due to drop evaporation or compression).
